# Discovery of a Mixed and Prodrug-Like Inhibition Mechanism for Phosphocoumarins and Phosphoquinolinones against Human Carbonic Anhydrases

**DOI:** 10.1021/acs.jmedchem.6c00915

**Published:** 2026-04-29

**Authors:** Alessio Nocentini, Simone Giovannuzzi, Vincenzo Alterio, Alessandro Bonardi, Rudolfs Barons, Raivis Zalubovskis, Wagdy M. Eldehna, Rossella Aronne, Davide Esposito, Enrico Luchinat, Giuseppina De Simone, Gianluca Bartolucci, Paola Gratteri, Mattia Mori, Claudiu T. Supuran

**Affiliations:** † NEUROFARBA Department, Section of Pharmaceutical Sciences, 9300 University of Florence, Sesto Fiorentino 50019, Italy; ‡ Institute of Biostructures and Bioimaging, 366187 National Research Council, Napoli 80145, Italy; § 187008 Latvian Institute of Organic Synthesis, Riga LC-1006, Latvia; ∥ Institute of Chemistry and Chemical Technology, Riga Technical University, Riga LV-1048, Latvia; ⊥ Department of Pharmaceutical Chemistry, Faculty of Pharmacy, 9313 Kafrelsheikh University, Kafrelsheikh 33516, Egypt; # Department of Biotechnology, Chemistry and Pharmacy, University of Siena, Siena 53100, Italy; ∇ CERM - Magnetic Resonance Center, University of Florence, Sesto Fiorentino 50019, Italy; ○ Department of Chemistry “Ugo Schiff”, University of Florence, Sesto Fiorentino 50019, Italy

## Abstract

Phosphocoumarins and a first-in-class unsubstituted phosphoquinolinone are disclosed as previously unrecognized carbonic anhydrase (CA) inhibitors, displaying multimodal inhibition within a tunable coumarin-like scaffold. Acidic phosphocoumarins display inhibition of physiologically relevant human CAs, particularly tumor-associated isoforms IX and XII (K_I_s: 0.08–0.28 μM) through a composite, two-step mechanism: the ligand first anchors the zinc-bound water molecule before displacing it to directly coordinate the catalytic zinc ion, without CA-mediated hydrolysis. Conversely, a methyl-ester phosphocoumarin functions as an isoform-selective prodrug, undergoing CA-mediated cyclic phosphoester hydrolysis to selectively generate a potent hCA IX/XII inhibitor (K_I_s: 54–62 nM), whereas the phosphoquinolinone acts as a direct binder (K_I_s: 0.18–0.29 μM vs hCA IX/XII). The complementary mechanisms are supported by QM/MM and long-time scale MD simulations, crystallographic studies, ^31^P NMR, HRMS, and MS/MS. Selected derivatives exhibit low-micromolar antiproliferative activity and induce apoptosis in cancer cells, fostering phosphorus-heterocycles as a mechanistically rich platform for isoform-selective CA inhibition and targeted drug design.

## Graphical abstract

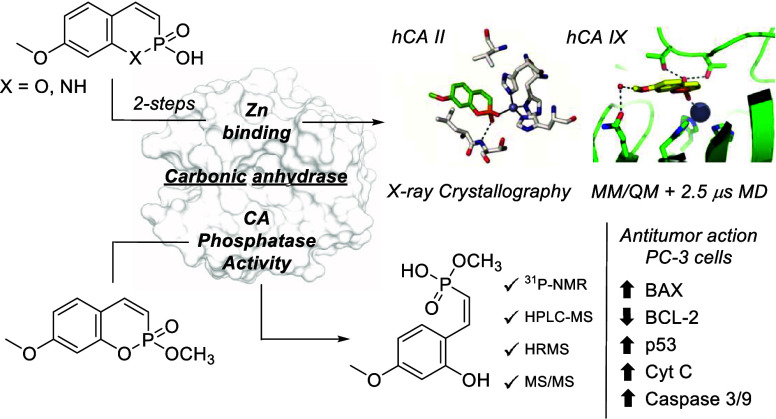

## Introduction

Carbonic anhydrases (CAs) are a family of zinc metalloenzymes that catalyze the reversible hydration of carbon dioxide to bicarbonate and a proton, a fundamental reaction to numerous physiological processes including respiration, pH regulation, and ion transport.^1^ CAs are validated targets for therapeutic intervention in various diseases such as glaucoma, epilepsy, obesity, and cancer.^2^ Traditional CA inhibitors such as sulfonamides, which bind the catalytic zinc ion by displacing the zinc-bound water molecule or hydroxide ion, have been extensively developed.^3^ Despite their efficacy, sulfonamides often exhibit poor isoform selectivity, paving the way to the exploration of novel chemotypes of CA inhibitors.^3^ Advances in the past decade have introduced coumarins and sulfocoumarins as new classes of CA inhibitors.^4,5^ In contrast to sulfonamides, coumarins act as prodrugs that are hydrolyzed by the esterase activity of CAs to form 2-hydroxy-cinnamic acids, which then bind at the entrance of the active site, leading to isoform-selective inhibition (Figure 1).^4^ Similarly, sulfocoumarins undergo CA-mediated hydrolysis to yield sulfonic acids that anchor the zinc-bound water molecule, resulting in CA inhibition.^5^ These findings showed the selectivity of prodrug-based CA inhibitors, for which we previously suggested a mechanism of action.^6^

**Figure 1:**
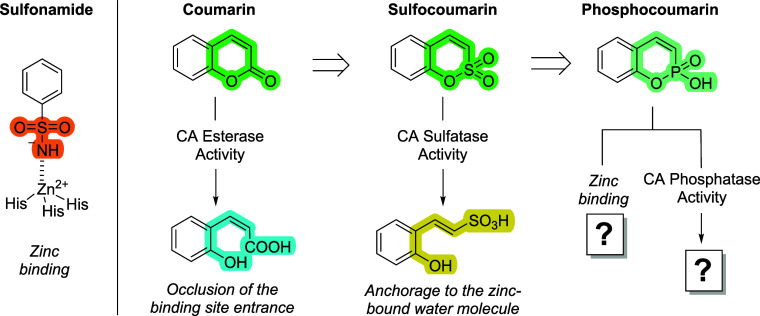
Design of phosphocoumarins as CA inhibitors.

We propose here an advancement in the field, i.e., phosphocoumarins as a novel CA inhibitor class, structurally mimicking coumarins/sulfocoumarins but with phosphorus replacing carbon or sulfur. Phosphorus (group V) imparts unique electronic properties to phosphocoumarins, conferring acidity and the potential to bind the catalytic zinc ion of CAs. In 2010, we demonstrated the phosphatase activity of α-CAs,^7^ while in 2007 the phosphonate foscarnet was crystallized in complex to hCA I, revealing a zinc-binding capability.^8^ Hence, phosphocoumarins might inhibit CAs both before and after hydrolysis, offering a composite and versatile mechanism of action. Moreover, in 2019 we described phosphonamidates, phosphorus-based sulfonamide analogs, suggesting that additional substitution at the zinc-binding group (ZBG) can extend enzyme interactions while coordinating catalytic zinc.^9^

Hence, we present the design, *ex novo* synthesis and characterization of the CA inhibition mechanism of phosphocoumarins/phosphoquinolinone. The compounds were evaluated for CA inhibition against a panel of isoforms at different times and thoroughly investigated with QM/MM methods and molecular dynamics (MD) simulations and X-ray crystallography. CA-mediated hydrolysis was studied by NMR and MS techniques. Finally, compounds displaying potent and selective inhibitory activity against the cancer-associated CA isoforms IX and XII were further assessed for their antitumor effects.

## Results and Discussion

### Chemistry

A convenient method for the synthesis of acidic/methyl ester phosphocoumarins, and the first phosphoquinolinone compound was first developed (Scheme 1). In fact, a literature survey highlighted a few papers reporting the synthesis of unsubstituted phosphocoumarins,^10,11^ but the synthesis of the key reagent **2** was not described and we first optimized the only synthetic procedure found in a 1990 patent.^12^ Acetaldehyde diethyl acetal **1** was treated with PCl_5_ in dichloromethane at reflux temperature and therefore with sodium dithionite (Scheme 1, main text). After filtering, the attained 2-ethoxyvinylphosphonic acid dichloroanhydride **2** was distilled. The latter reacted with phenol derivatives **3** or **4** in toluene in the presence of triethylamine to give compounds **5** and **6**, showing a chemical shift of the phosphorus nucleus from 4.57 to 7.27 and 8.11 ppm in the ^31^P NMR spectrum, respectively (Scheme 1, main text). Heating chlorides **5** and **6** in 1,4-dioxane in the presence of trifluoroacetic acid results in oxaphosphorinine **7** and **8** (Scheme 1, main text). The ring closure and aromatization of compound **7** were accompanied by characteristic chemical shift changes in both ^1^H NMR and ^31^P NMR spectra (Supporting Information). In the ^1^H NMR spectrum of compound **7**, the double bond protons appear as characteristic doublets of doublets at δ 6.16 ppm (^2^ *J*_*H–P*_ = 19.7 Hz) and δ 7.38 ppm (^3^ *J*_*H–P*_ = 42.4 Hz), while the aromatic protons resonate as doublets at δ 6.79 ppm (meta) and δ 7.42 ppm (ortho). The signal at δ 6.16 ppm (vs 5.34 of precursor **2**) reflects sp^2^ hybridization and aromatic deshielding, and a geminal ^2^ *J*_*H–P*_ coupling (19.7 Hz) matches the precursor **2** (21.8 Hz) and typical vinylic H–P values (15–25 Hz). The ^31^P NMR signal undergoes dramatic deshielding reversal: from δ 34.55 ppm (compound **2**, acyclic P­(V)-OR_3_) to δ 5.83 ppm (compound **7**, cyclic P–O–C­(sp^2^)). These diagnostic NMR signatures provide structural confirmation of phosphocoumarin formation and were consistently observed for analogues **8** and **13**. Starting from compound **7**, two other derivatives were attained by (i) demethylation with BBr_3_ in dichloromethane to give the phenol phosphocoumarin **9**; (ii) phosphonic acid esterification by chlorination in SOCl_2_ and successive reaction with MeOH to achieve methyl ester **10** as a racemic mixture (Scheme 1). To synthesize the phosphoquinolinone **13**, reagent **2** was treated with aniline **11** achieving phosphonamidic chloride **12**, that was thus cyclized by treatment with TFA in 1,4-dioxane (Scheme 1). Yields of the overall process to achieve the phosphoquinolinone **13** were significantly lower compared to the procedure leading to phosphocoumarin **7** and **8**.

**Scheme 1:**
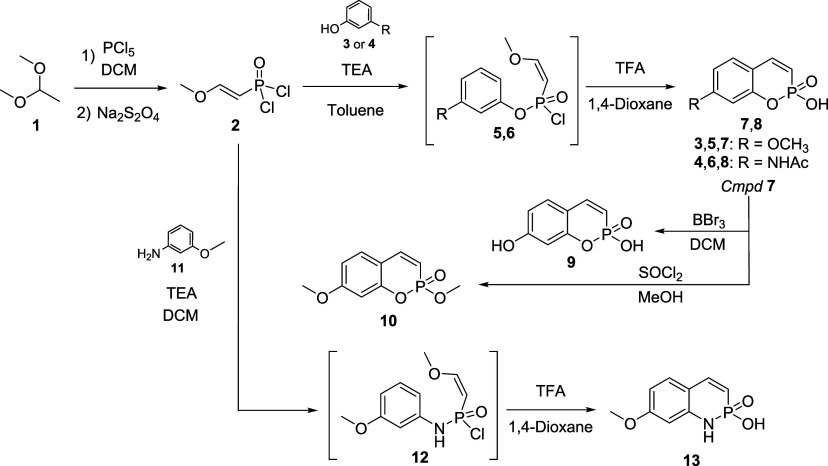
Synthetic Pathway to Phosphocoumarins **7**–**10** and Phosphoquinolinone **13**

### Enzyme Inhibition

Compounds **7–10** and **13** were tested against a panel of relevant human CA isoforms (Table 1). The data were obtained by a stopped-flow technique monitoring CO_2_ hydration to bicarbonate and protons catalyzed by CAs.^13^ With canonical CA inhibitors (CAIs), (e.g., sulfonamides, dithiocarbamates, etc.) 15 min incubation time is sufficient for the formation of the enzyme–inhibitor adduct and the detection of the highest inhibition rate. In contrast, when prodrug CA inhibitors are tested (e.g., coumarins and sulfocoumarins) an incubation of approximately 6 h is required.^4^ Given the potential hybrid nature of the phosphocoumarin, the effect of the incubation time on CA inhibition by **7–10** and **13** was investigated. K_I_s were thus measured after 15 min, 1, 2, 4, 6, and 12 h of incubation. The inhibitory effects of compounds over time reveal distinct mechanistic behaviors. K_I_ values decreased from 15 min to 1 h incubation for compounds **7–9**, with no further reduction thereafter. This suggests a time-dependent inhibition process where the inhibitor progressively binds within the enzyme active site, likely undergoing conformational adjustments or chemical transformations, enhancing affinity over time. The inhibitory activity reaches single-digit nanomolar or submicromolar K_I_s against hCA I, II, and IV, while achieving mid-to-low nanomolar potency against hCA IX and XII. Instead, phosphoquinolinone **13** exhibits a different pattern, with K_I_ values (that mirror the trend of phosphocoumarins **7–9** after 1 h) remaining relatively stable across different incubation times, with nonsignificant change from 15 min to 1 h. This indicates that **13** acts through a direct mechanism that does not involve conformational adjustments or chemical transformations. Conversely, compound **10** demonstrated a marked time dependency, with initial K_I_ values >100 μM at 15 min and further reductions from 1 to 6 h (and no further reduction thereafter) up to nanomolar concentrations for tumor-associated isoforms hCA IX and XII (Table 1). This trend suggests that **10** might act through a prodrug-like or slow-activation mechanism, consistent with that of nonacidic coumarin derivatives,^4^ where the inhibitor requires time to achieve its maximum inhibitory effect. Overall, the data show that incubation time critically influences the measured CA inhibition potency and selectivity profiles, with important mechanistic implications for the further development and biochemical characterization of this novel chemotype of CAs inhibitors.

**Table 1:** Inhibition Data of Human CAs I, II, IV, IX, XII with Compounds **7–10** and **13** by a Stopped Flow Assay Method Using **AAZ** as a Standard Inhibitor

	K_I_ (μM)^a^				
Cmpd	hCA I	hCA II	hCA IV	hCA IX	hCA XII
*15 min incubation*					
**7**	>100	25.1 ± 2.0	22.3 ± 1.8	3.4 ± 0.3	9.0 ± 0.8
**8**	49.7 ± 3.2	30.4 ± 2.6	10.4 ± 1.2	4.6 ± 0.3	7.5 ± 0.6
**9**	>100	15.6 ± 1.2	12.9 ± 1.1	1.7 ± 0.2	6.1 ± 0.6
**10**	>100	>100	>100	>100	>100
**13**	4.7 ± 0.2	4.4 ± 0.3	5.2 ± 0.4	0.29 ± 0.03	0.17 ± 0.02
*1 h incubation*					
**7**	8.5 ± 0.5	2.5 ± 0.2	4.1 ± 0.3	0.28 ± 0.02	0.19 ± 0.01
**8**	5.3 ± 0.4	1.8 ± 0.2	1.1 ± 0.1	0.13 ± 0.02	0.081 ± 0.007
**9**	6.3 ± 0.5	0.98 ± 0.8	1.5 ± 0.2	0.22 ± 0.03	0.13 ± 0.01
**10** ^b^	>100 (79.3 ± 6.2)	>100 (52.1 ± 4.1)	73.6 ± 6.5 (3.6 ± 0.3)	1.2 ± 0.1 (0.054 ± 0.004)	5.3 ± 0.4 (0.062 ± 0.006)
**13**	4.2 ± 0.3	3.9 ± 0.4	5.5 ± 0.5	0.29 ± 0.02	0.18 ± 0.02
**AAZ**	0.25	0.012	0.074	0.025	0.003

a Inhibition data are expressed as means ± SEM of three different assays.

b 6 h incubation in brackets.

### Computational Study

Using the **7**/hCA IX complex as prototype, the inhibition mechanism of phosphocoumarins was investigated *in silico* by coupling docking and MD simulations with QM/MM calculations (Figure 2). Initially, two docking options were used: (i) fixed Zn-binding water molecule (option water “on” in GOLD), and (ii) displaceable Zn-binding water molecule (option water “Toggle” in GOLD). Based on binding mode and—especially—on the comparison of the dimensionless ChemPLP score, we envisaged that the most effective binding mode of **7** to hCA IX is established by direct coordination of the catalytic Zn­(II) ion by the molecule, upon displacement of the Zn-bound water molecule (Table S1).

**Figure 2:**
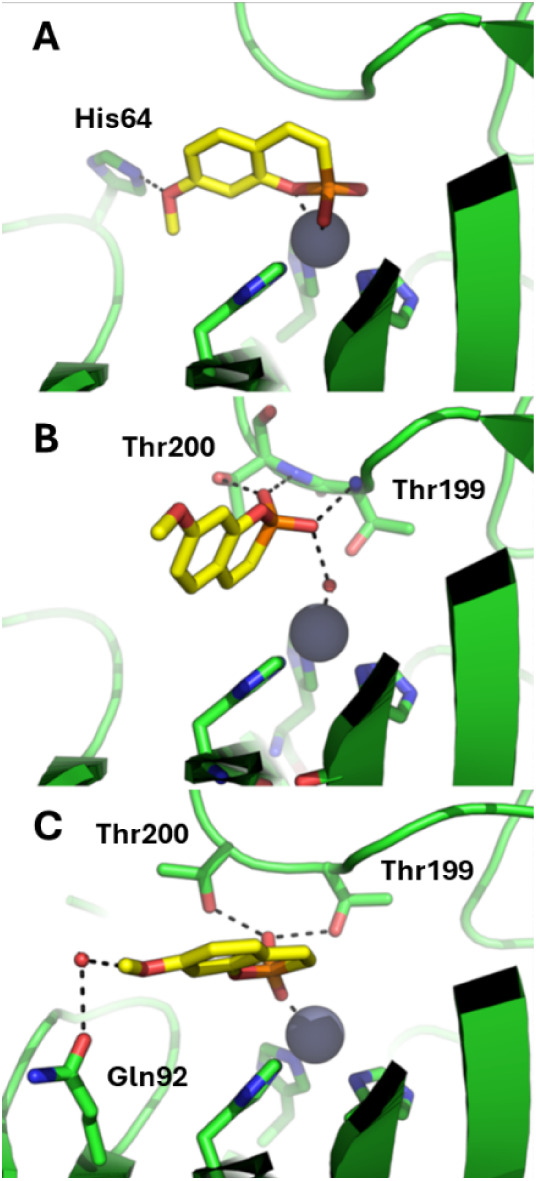
Representative poses of **7** bound to hCA IX (PDB 5FL4) as obtained by A) molecular docking simulations; B) intermolecular recognition by 500 ns of MD simulations; C) extension of MD simulations up to 2.5 μs. hCA IX is shown as green cartoon, residues interacting with **7** and zinc-binding histidine residues are shown as sticks. Compound **7** is shown as yellow sticks. The catalytic zinc ion is shown as a gray sphere. H atoms were omitted; water molecules bridging **7** to hCA IX are shown as small red spheres. Polar interactions are highlighted by black dashed lines.

The MD-based recognition experiment between **7** and hCA IX showed that the small molecule has a remarkable affinity for the catalytic site of hCA IX, which is reached quite early in the molecular simulation. Most notably, the interaction of **7** within the hCA IX catalytic site is stable in the simulation time, with the molecule failing to come back to the solvent up to 2.5 μs of MD simulation (Figure 2). Cluster analysis was then carried out on MD frames to understand structural determinants that regulate the recognition and binding of **7** to hCA IX. Specifically, frames of the first 500 ns of MD simulations were first analyzed, showing that **7**—upon recognizing the hCA IX catalytic site—binds preferentially the metal center by anchoring the Zn-bound water molecule. Cluster analysis of the frames composing the subsequent 2 μs of MD simulations showed that **7** displaces the Zn-bound water molecule to directly coordinate the catalytic Zn­(II) ion. Besides Zn­(II) coordination, **7** establishes two H-bonds with the side chain of Thr199 and Thr200. Finally, a water-bridged H-bond interaction with Gln92 further reinforces the interaction of the phosphocoumarin derivative to the catalytic site of hCA IX. Thus, in the recognition step compound **7** anchors the Zn-binding water molecule and it is endowed with a moderate affinity for hCA IX, such as observed for many different chemotypes.^3^ Extending the simulation time up to 2.5 μs showed that **7** can displace the Zn-bound water molecule and anchor directly the catalytic Zn­(II) ion, thus exerting a strong inhibition of the enzyme.

The hCA IX/**7** complex obtained after 500 ns of MD simulations (*i.e.*, **7** anchored to the Zn-bound water molecule) was divided into a HL treated at the QM level and a LL treated at the MM level. To investigate the possibility that **7** is hydrolyzed by the hCA IX catalytic site, in agreement with a previous study on a parent sulfocoumarin derivative, a similar computational setting was used herein. The published action mechanism for sulfocoumarin consists of four subsequent steps: (i) opening of the sulfocoumarin cycle elicited by the nucleophilic attack from the Zn-bound hydroxide ion toward the sulfonic ester; (ii) Z/E isomerization of the cycle’s double bond; (iii) the displacement of the Zn-bound ligand by a molecule of water from the solvent; (iv) release of the sulfonate derivative and restoration of the catalytically competent form of the hCA IX active site. Since we were able to reproduce the sulfocoumarin hydrolysis, the settings were applied to compound **7** by selecting the distance between the P and the cyclic O atoms as redundant coordinate and using B3LYP/3-21g* level of theory and basis set. While the reactant complex of **7** with the hCA IX converged in the same way as the reference sulfocoumarin, the TS never converged, and we did not observe ring opening and double bond isomerization. To rule out this issue, multiple simulations were run: (i) using different functional (i.e., M06–2x and B3LYP); (ii) using different basis sets (3-21g and 6-31g*); (iii) scanning different reaction coordinates (P–O distance, P–O_WAT_ distance, and the C–C=C–P dihedral angle); (iv) using a water molecule as a nucleophile instead of the OH-group. Unfortunately, none of these calculations yielded a converged stationary point. Finally, we manually designed the TS of **7** in a ring-opened form, but the optimization step ended with the native closed form of **7**, suggesting that—despite chemical similarity—the phosphocoumarin **7** cannot be hydrolyzed by the catalytic site of hCA IX in simulated conditions such as previously observed for a sulfocoumarin derivative. Hence, the binding mode predicted by MD simulations, coupled with QM/MM evidence that **7** is not hydrolyzed by hCA IX, can explain the inhibition kinetics experimentally observed.

### X-ray Crystallography

Further confirmation of the proposed inhibition mechanism was achieved through crystallographic investigations. The crystal structure of hCA II in complex with **7** was determined at a resolution of 1.65 Å by soaking the inhibitor into preformed crystals of hCA II, selected as a model isoform for these studies. After the initial stages of refinement, inspection of |Fo-Fc| and |2Fo-Fc| electron density maps revealed the presence of a single inhibitor molecule in the enzyme active site. Consistent with the QM/MM calculations and MD simulations, the inhibitor binds to the enzyme in its nonhydrolyzed form by displacing the zinc-bound water molecule and coordinating the metal ion through one of its exocyclic oxygen atoms (Figure 3A). The binding is further stabilized by a hydrogen bond between the second exocyclic oxygen and the backbone amide nitrogen of Thr199 (Figure 3B). The bicyclic ring is positioned in the middle of the active site, where it contributes to complex stabilization through hydrophobic interactions with the side chains of Val121, Phe131, and Leu198.

**Figure 3:**
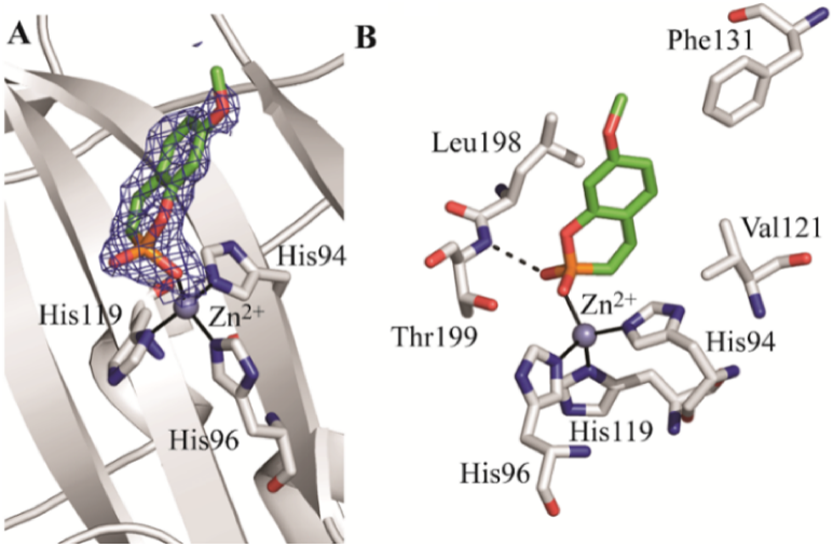
X-ray crystal structure of the hCA II/**7** complex. (A) Active site region: σA-weighted (|2Fo-Fc|, φc) map (contoured at 1.0σ) relative to the ligand is shown. (B) Details of the interactions established by 7 within the enzyme active site. Residues involved in hydrogen bonds and hydrophobic interactions (*d* < 4 Å) are shown. Continuous lines indicate zinc ion coordination, whereas dashed lines indicate hydrogen bond distances (*d* < 3.2 Å).

Figure S1 (Supporting Information) shows the structural superposition of the crystallographic structure of the hCA II/**7** complex and the hCA IX/**7** complex obtained by MD simulations. Although in both cases the compound directly coordinates to the catalytic zinc ion, differences in the orientation of the bicyclic ring are observed that can be due to subtle variations in the active sites of hCA II and hCA IX and/or to the different techniques used to obtain the results. This binding variance likely accounts for the differing inhibition potency between hCA IX and hCA II. A comparison of the binding mode of **7** within the CA active site with those of its structural analogues (hydrolyzed coumarin and sulfocoumarin, and 2-thioxocoumarins)^4,5^ is depicted in Figure S2, Supporting Information.

The comparison of the binding mode of **7** within the CA active site with those of its structural analogues (hydrolyzed coumarin and sulfocoumarin, and 2-thioxocoumarins)^3–5^ reveals significant differences (Figure S2). Indeed, unlike coumarins and sulfocoumarins, which undergo enzymatic hydrolysis and bind in their hydrolyzed forms through a suicide inhibition mechanism, **7** binds in its intact form. Upon hydrolysis, sulfocoumarins anchor to the ZBW molecule (Figure S2C), whereas coumarins bind at the entrance of the active site (Figure S2D), effectively blocking substrate access. In contrast, 2-thioxocoumarins do not undergo hydrolysis, but anchor to the ZBW molecule similarly to sulfocoumarins (Figure S2E).

### ^31^P NMR Study

To further elucidate the phosphocoumarin mechanism of action, we set up and conducted a ^31^P NMR study to assess the ligand hydrolysis upon phosphoesterase activity of hCA I, II, IX, and XII. Phosphocoumarin **7** and its methyl ester analog **10** were used. The ^31^P NMR spectra of each compound were first recorded in the test conditions in absence of the enzyme to identify the phosphorus signal of the free ligand and were subsequently repeated at different incubation times (15 min, 1 h, 2 h, 4 h, 6 h) in the presence of the enzymes to monitor the appearance of new phosphorus-containing species, indicative of phosphoester bond cleavage. The results revealed a clear difference between the two compounds. The ^31^P NMR resonance of acidic phosphocoumarin **7** remained unchanged throughout the incubation period, and no additional signals were detected in the presence of any CA isoform (Figure S3, Supporting Information), confirming that the phosphoester group of **7** is not hydrolyzed by CAs, consistent with the *in silico* and X-ray data. In contrast, the methyl ester derivative **10** showed time-dependent spectral changes in the presence of the enzymes. A new phosphorus signal progressively appeared during incubation, with variable outcome depending on the CA isoform (spectra after 1 h shown in Figure S4, Supporting Information). The process was most pronounced with hCA IX, where after approximately 6 h of incubation (Figure 4), the original signal of compound **10** was almost completely replaced by a new peak, suggesting the enzyme-promoted conversion to a hydrolyzed species (hereafter indicated as compound **10′**). Formation of the same new signal was also observed with CA XII, albeit at a slower rate, whereas only minor or changes occurred with hCA I and II within the same time frame. Different from the acidic phosphocoumarin **7**, these findings confirm that the methyl ester analog **10** can undergo isoform-dependent hydrolysis, particularly with tumor-associated isoforms hCA IX and XII, supporting the prodrug-like mechanism proposed on the basis of kinetic data.

**Figure 4:**
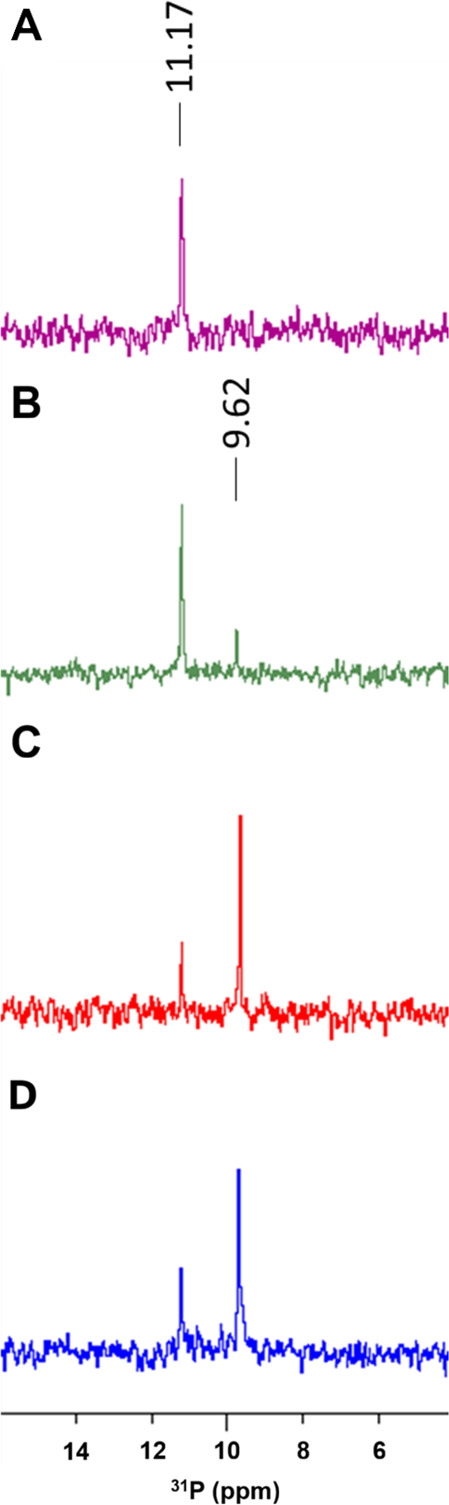
^31^P NMR spectra of compound **10** after a 6 h incubation with hCAs A) I, B) II, C) IX, and D) XII. The original phosphorus resonance corresponding to the intact methyl ester phosphocoumarin is shown alongside the new resonance peak indicative of the hydrolyzed phosphoester species.

### Mass Spectrometry

To identify compound **10′**, predominantly formed upon 6 h enzymatic incubation of compound **10** with hCA IX, a series of mass spectrometry (MS) experiments was carried out, including tandem mass spectrometry (MS/MS) and high-resolution mass spectrometry (HRMS). At first, HPLC–MS analysis of compound **10** confirmed the presence of a single chromatographic component (Figure S5A, Supporting Information). The corresponding mass spectrum displayed one intense signal at *m*/*z* 227 (Figure S5B, Supporting Information), assigned to the protonated molecular ion [M + H]^+^. HRMS analysis of this peak (Figure S5C, above) revealed a single signal at *m*/*z* 227.04672, consistent with the molecular formula C_10_H_12_O_4_P (mass error = –0.23 ppm).

Thus, HPLC–MS analysis of the incubation mixture revealed the disappearance of the peak corresponding to the parent compound (detailed analytical data of compound **10** in the Supporting Information) and the appearance of two main new chromatographic signals (Figure 5A). The most abundant component, eluting at RT = 5.40 min, exhibited an MS spectrum with an intense ion at *m*/*z* 245 (Figure 5B). When assigned to the [M + H]^+^ species, this +18 Da shift compared with compound **10** suggested the incorporation of a water molecule (compound **10′**, Figure 5B). HRMS analysis of the same peak confirmed an elemental composition corresponding to C_10_H_14_O_5_P (*m*/*z* 245.05730, mass error = –0.15 ppm), thus differing from the parent compound by one H_2_O unit (Supporting Information, Figure S5).

**Figure 5:**
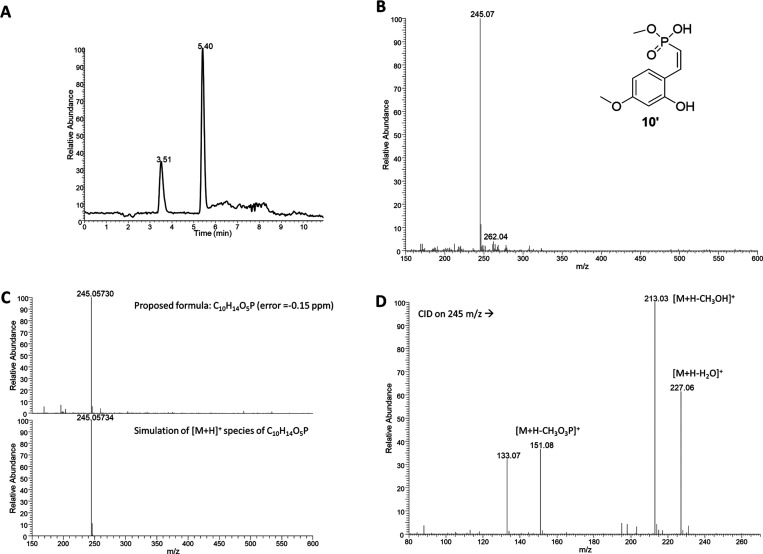
A) HPLC–MS profile of compound **10** after incubation with hCA IX; B) MS spectrum positive ions of peak at RT = 5.40 min; C) Comparison between HRMS (30k FWHM) positive ions spectrum of peak at RT = 5.40 min and the simulation of the proposed formula; D) MS/MS spectrum of compound **10′** obtained upon incubation of compound **10** with hCA IX.

To further confirm the structural assignment, HPLC–MS/MS analysis of the incubation solution was performed by selecting the ion at *m*/*z* 245 as precursor (Figure 5D). The spectrum displayed fragments at *m*/*z* 227 (−18 Da, water loss), *m*/*z* 213 (−32 Da, methanol loss), and *m*/*z* 151 (−94 Da, methyl phosphonate loss). The combined MS, MS/MS, and HRMS data clearly indicate that compound** 10** undergoes enzyme-dependent hydrolysis by hCA IX, yielding compound **10′**. No analogous products were detected in the control samples lacking the enzyme.

### Antitumor Study

The antiproliferative activity of phosphocoumarins **7–10** and phosphoquinolinone **13** was evaluated across a panel of human cancer cell lines, including breast carcinoma (MCF-7 and MDA-MB-231), colorectal adenocarcinoma (HCT116), and prostate carcinoma (PC-3) using the MTT assay (Table 2). Staurosporine (STA) and doxorubicin (DOX) served as positive control.

**Table 2:** *In Vitro* Anti-Proliferative Activity of **7–10** and **13** against MCF-7, MDAMB-231, HCT116, and PC-3 Cancer Cell Lines

	IC_50_ (μM)^a^			
Cmpd	*MCF-7*	*MDA-MB-231*	*HCT116*	*PC-3*
**7**	13.4 ± 0.56	10.2 ± 0.65	0.69 ± 0.02	1.05 ± 0.06
**8**	48.6 ± 1.2	22.0 ± 1.0	8.33 ± 0.34	5.78 ± 0.32
**9**	4.61 ± 0.23	20.9 ± 0.9	2.57 ± 0.08	2.62 ± 0.11
**10**	16.1 ± 0.71	4.17 ± 0.32	2.02 ± 0.09	1.83 ± 0.18
**13**	5.16 ± 0.20	18.0 ± 0.81	0.95 ± 0.06	2.74 ± 0.12
**STA**	6.99 ± 0.32	10.6 ± 0.51	8.86 ± 0.33	4.91 ± 0.17
**DOX**	1.80 ± 0.41	3.62 ± 0.32	0.82 ± 0.03	0.56 ± 0.02

a IC_50_ values are the mean ± SD of three separate experiments.

PC-3 cells exhibited highest sensitivity in antiproliferative assays (mean IC_50_ = 2.78 μM), prompting a further evaluation of compounds **7**, **10**, and **13** for apoptotic effects at their respective IC_50_ concentrations. Derivatives **7**, **10**, and **13** significantly shifted the Bax/Bcl-2 axis, upregulating Bax expression to 245.3 pg/mg (6.0-fold), 289.1 pg/mg (7.1-fold), and 231.7 pg/mg (5.7-fold), respectively (Figure 6). Concurrently, Bcl-2 was potently downregulated to 2.075 ng/mg (63.6% reduction), 3.192 ng/mg (43.9% reduction), and 2.763 ng/mg (51.5% reduction). To confirm an intrinsic apoptotic pathway activation, the effect of derivatives **7**, **10**, and **13** toward key effectors in PC-3 cells was assessed (Figure 6C–F). Caspase-3 (executioner) levels increased to 322.7 ng/mg (5.3-fold), 278.6 ng/mg (4.6-fold), and 302.8 ng/mg (5.0-fold), while caspase-9 (initiator) reached 21.48 ng/mg (14.4-fold), 18.85 ng/mg (12.6-fold), and 15.7 ng/mg (10.5-fold) versus controls. The robust caspase-9 to caspase-3 cascade (max 14.4-fold activation) corroborates the execution of the mitochondrial apoptotic pathway downstream, that could be related to CA-mediated pH dysregulation. Compounds **7**, **10**, and **13** also triggered cytochrome c release into PC-3 cell cytosol at 0.35 ng/mg (4.4-fold), 0.48 ng/mg (6.0-fold), and 0.40 ng/mg (5.0-fold) versus controls. Additionally, p53 upregulation reached 787.3 ng/mg (10.9-fold), 637.8 ng/mg (8.8-fold), and 586.8 ng/mg (8.1-fold), respectively, amplifying Bax activation and Bcl-2 suppression.

**Figure 6:**
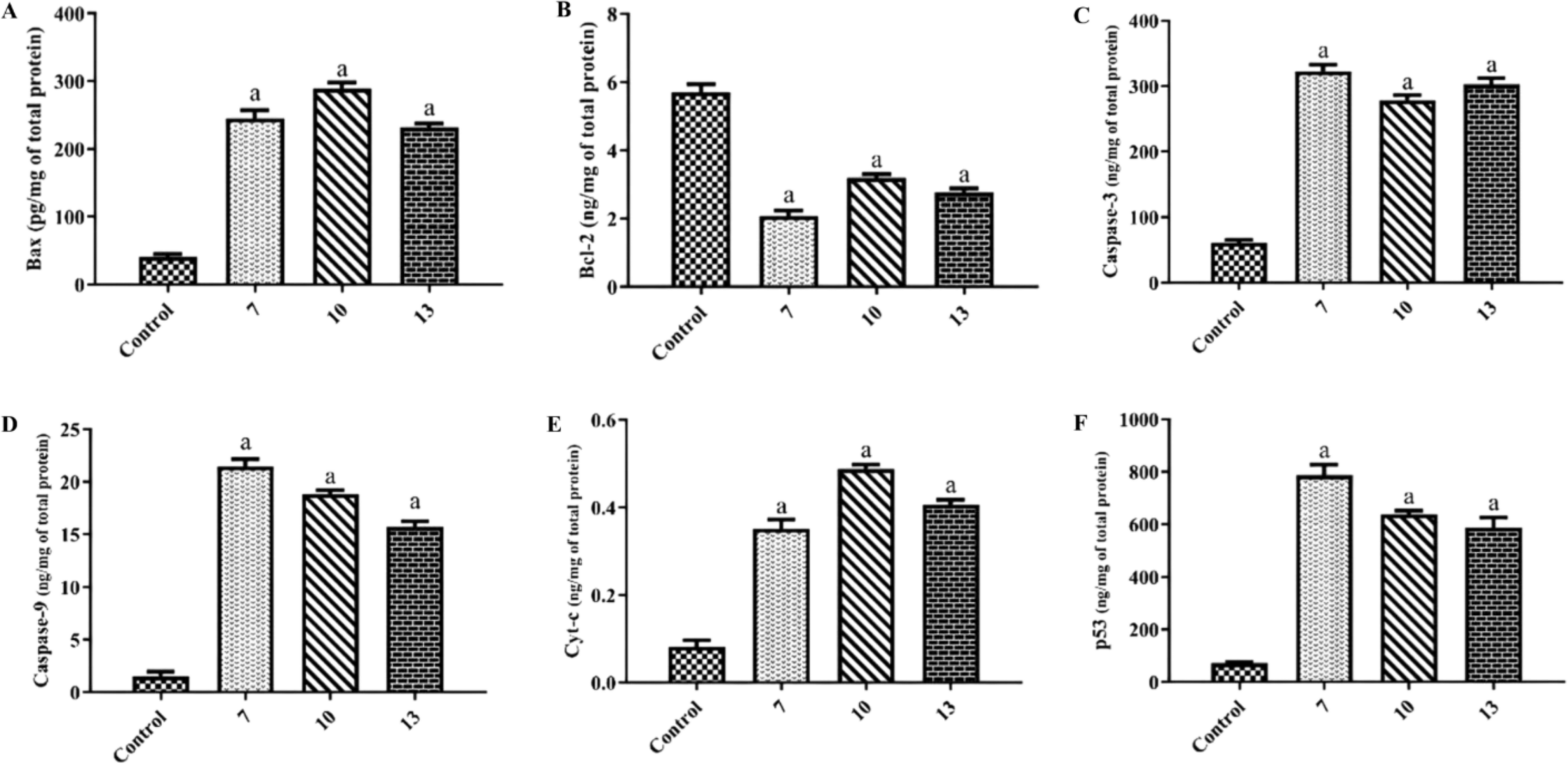
Effect of compounds **7**, **10**, and **13** on the protein levels of Bax (A), Bcl-2 (B), caspase-3 (C), caspase-9 (D), cytochrome c (Cyt-c) (E), and p53 (F) in prostate PC-3 cells treated with the ligands at their IC_50_ concentrations compared with control (DMSO). Data are presented as mean ± SD. Statistical analysis was performed using one-way ANOVA followed by Tukey’s multiple comparisons test; ^a^ indicates a significant difference compared with control (*p* < 0.05).

## Conclusions

This study establishes phosphocoumarins/phosphoquinolinones as multimodal CA inhibitors. Acidic phosphocoumarins (compounds **7**, **8**, and **9**) act as time-dependent inhibitors that follow a two-step coordination process. *In silico*, X-ray crystallographic and NMR data demonstrate that these ligands do not undergo zinc-mediated hydrolysis. Instead, in their deprotonated form they first anchor the zinc-bound water/hydroxide ion and only subsequently displace it to directly coordinate the catalytic zinc ion. Instead, primary sulfonamides, weak acids, first undergo deprotonation by the zinc-bound hydroxide before rapidly displacing zinc-bound water (maximal effect at 15 min). The phosphocoumarin methyl ester derivative **10** behaves as a prodrug-like inhibitor, closely mirroring coumarins/sulfocoumarins: ^31^P NMR and MS analyses show that **10** undergoes isoform-dependent hydrolysis, predominantly by the tumor-associated isoforms hCA IX and XII, to generate a more effective, phosphonate inhibitor. A hydrolysis mechanism analogous to that we previously described for sulfocoumarins^6^ can therefore be proposed. The phosphoquinolinone **13** displays the highest inhibition after 15 min, consistent with a direct binding mode that does not require either hydrolysis or slow binding rearrangements. Compounds **7**, **10**, **13** exhibit notable antiproliferative activity across a panel of human cancer cell lines, triggering the intrinsic mitochondrial apoptotic pathway, as evidenced by p53 and Bax upregulation, Bcl-2 downregulation, cytochrome c release, and robust activation of the caspase-9/caspase-3 cascade.

## Methods

### Chemistry

Anhydrous solvents and all reagents were purchased from Merck Sigma, TCI, and Fluorochem. All reactions involving air- or moisture-sensitive compounds were performed under a nitrogen atmosphere using dried glassware and syringes techniques to transfer solutions. Nuclear magnetic resonance (^1^H NMR, ^13^C NMR, ^31^P NMR) spectra were recorded using a Bruker Advance III 400 MHz spectrometer in CDCl_3_ and DMSO-*d*_6_. Chemical shifts are reported in parts per million (ppm) and the coupling constants (J) are expressed in Hertz (Hz). Splitting patterns are designated as follows: s, singlet; d, doublet; t, triplet; q, quartet; m, multiplet; dd, doublet of doublets. The assignment of exchangeable protons (OH and NH) was confirmed by the addition of D_2_O. Analytical thin-layer chromatography (TLC) was carried out on Merck silica gel F-254 plates. Flash chromatography purifications were performed on Merck Silica gel 60 (230–400 mesh ASTM) as the stationary phase and ethyl acetate/*n*-hexane or methanol/dichloromethane were used as eluents. Melting points (mp) were measured in open capillary tubes with a Gallenkamp MPD350.BM3.5 apparatus and are uncorrected. High-resolution mass spectrometry (HRMS) analyses were performed using a Thermo Finnigan LTQ Orbitrap mass spectrometer equipped with an IonMax electrospray ionization (ESI) interface and coupled to a Dionex Ultimate 3000 HPLC system, consisting of a ternary gradient pump, an autosampler, a column oven, and a UV–Vis detector. Stock solutions of each analyte were prepared at a concentration of 1 mg mL^–1^ in methanol containing 10% DMSO. Working solutions (10 mg L^–1^) were obtained by diluting the stock solutions with acetonitrile/mQ water (1:1, v/v). Accurate mass-to-charge (*m*/*z*) measurements were carried out by direct infusion of the working solutions using a syringe pump at a flow rate of 10 μL min^–1^, and signals were acquired in either positive or negative ion mode. The ESI source parameters were as follows: needle voltage, 4 kV; capillary voltage, 13 V; tube lens voltage, 60 V; capillary temperature, 290 °C. Nitrogen was used as sheath, auxiliary, and sweep gas, set at 15, 8, and 0 arbitrary units (a.u.), respectively. Under these experimental conditions, protonated or deprotonated molecular ions ([M + H]^+^ or [M–H]^−^) of the studied compounds were monitored. Spectra were acquired with an appropriate dwell time to achieve a resolution of 60,000 (full width at half-maximum, fwhm). The elemental composition of each compound was determined based on the measured accurate *m*/*z* values, accepting only assignments with a mass error below 2.0 ppm and a noninteger ring double bond equivalent (RDB) value, in order to consider only even-electron ion species.^14^ Purity was assessed under the same chromatographic conditions using a UV–Vis detector monitored at 280 ± 10 nm, with analyte solutions prepared at 50 mg/L, confirming a >95% purity for all compounds studied in cell.

### Synthesis of (*E*)-(2-Methoxyvinyl)­phosphonic Dichloride (**2**)

1,1-Dimethoxyethane (2 g, 1.0 equiv) was added dropwise to a solution of PCl_5_ (3.0 equiv) in DCM (14 mL) cooled at 10 °C. The reaction mixture was stirred for 2 h at rt and then at 40 °C for 4 h. Thus, Na_2_S_2_O_4_ (2.0 equiv) was added portionwise, the suspension was stirred for 30 min and filtered. The solution was distilled to obtain **2** as a clear yellow oil. Yield 42%; ^1^H NMR (400 MHz, CDCl_3_): δ 3.78 (s, 3H, OC*H*_3_), 5.34 (dd, ^2^ *J*_*H–P*_ = 21.8 Hz, ^3^ *J*_*H–H*_ = 13.3 Hz, 1H, C*H*), 7.48 (dd, ^3^ *J*_*H–P*_ = 13.8, ^3^ *J*_*H–H*_ = 13.3 Hz, 1H, C*H*); ^31^P NMR (162 MHz, CDCl_3_): δ 34.55.

### Synthesis of 7-Substituted Phosphocoumarins **7** and **8** and Phosphoquinoline (**13**)

Phenol compounds **3** and **4** or aniline **11** (1.0 equiv) was added to a solution of **2** (2 g, 1.2 equiv) and TEA (1.5 equiv) in toluene (10 mL) cooled at 0 °C, and the reaction mixture was stirred at rt for 4 h. After monitoring by TLC (MeOH/DCM 10%). The suspension was filtered and the solvent was removed under vacuum to obtain **5**, **6** and **12**, used in the subsequent step without further purification.

Trifluoroacetic acid (1.1 equiv) was added to a solution of **5, 6** or **12** (1.0 g, 1.0 equiv) in 10 mL dioxane and the reaction mixture was stirred to reflux temperature for 10 h. The solution was cooled to r.t. and the obtained crystals were filtered, washed with ethanol, dried and the crude products were purified by flash chromatography (MeOH/DCM 5 to 10%) to give **7**, **8** and **13**.

### 2-Hydroxy-7-methoxybenzo­[e]­[1,2]­oxaphosphinine 2-Oxide (**7**)

Yield 42%; ^1^H NMR (400 MHz, DMSO-*d*_6_): δ 3.83 (s, 3H, OC*H*_3_), 6.16 (dd, ^2^ *J*_*H–P*_ = 19.7 Hz, ^3^ *J*_*H–H*_ = 12.4 Hz, 1H, C*H*), 6.79 (m, 2H, 2 x Ar-*H*), 7.38 (dd, ^3^ *J*_*H–P*_ = 42.4 Hz, ^3^ *J*_*H–H*_ = 12.4 Hz, 1H, C*H*), 7.42 (d, ^3^ *J*_*H–H*_ = 9.2 Hz, 1H, Ar-*H*); ^13^C NMR (100 MHz, DMSO-*d*_6_): δ 56.13, 103.96 (d,^3^ *J*_*C–P*_ = 6.8 Hz), 110.44, 112.34 (d,^1^ *J*_*C–P*_ = 167.5 Hz), 114.64 (d,^2^ *J*_*C–P*_ = 20.0 Hz), 131.21 (d,^4^ *J*_*C–P*_ = 1.1 Hz), 141.82, 153.18 (d,^3^ *J*_*C–P*_ = 7.7 Hz), 161.63 (d,^4^ *J*_*C–P*_ = 1.5 Hz); ^31^P NMR (162 MHz, DMSO-*d*_6_): δ 5.83; HRMS (*m*/*z*): calcd for C_9_H_8_O_4_P ([M–H]^−^), 211.0166; found, 211.0160.

### *N*-(2-Hydroxy-2-oxidobenzo­[e]­[1,2]­oxaphosphinin-7-yl)­acetamide (**8**)

Yield 28%; ^1^H NMR (400 MHz, DMSO-*d*_6_): δ 2.10 (s, 3H, C*H*_3_), 6.20 (dd, ^2^ *J*_*H–P*_ = 19.5 Hz, ^3^ *J*_*H–H*_ = 12.5 Hz, 1H, C*H*), 7.31 (dd, ^3^ *J*_*H–H*_ = 8.4 Hz, ^4^ *J*_*H–H*_ = 1.9 Hz, 1H, Ar-*H*), 7.32 (d, ^3^ *J*_*H–P*_ = 41.8 Hz, ^3^ *J*_*H–H*_ = 12.5 Hz, 1H, C*H*), 7.39 (d, ^3^ *J*_*H–H*_ = 8.4 Hz, 1H, Ar-*H*), 7.51 (d, ^4^ *J*_*H–H*_ = 1.9 Hz, 1H, Ar-*H*), 10.21 (s, 1H, exchange with D_2_O, CON*H*); ^13^C NMR (100 MHz, DMSO-*d*_6_): δ 24.61, 108.46 (d,^3^ *J*_*C–P*_ = 7.1 Hz), 113.66 (d,^1^ *J*_*C–P*_ = 167.5 Hz), 114.10, 116.48 (d,^2^ *J*_*C–P*_ = 19.7 Hz), 130.54, 141.60, 141.78, 152.13 (d,^3^ *J*_*C–P*_ = 7.9 Hz), 169.29; ^31^P NMR (162 MHz, DMSO-*d*_6_): δ 5.04; HRMS (*m*/*z*): calcd for C_10_H_9_NO_4_P ([M–H]^−^), 238.0275; found, 238.0269.

### 2-Hydroxy-7-methoxy-1H-benzo­[e]­[1,2]­azaphosphinine 2-Oxide (**13**)

Yield 20%; ^1^H NMR (400 MHz, DMSO-*d*_6_): δ 3.77 (s, 3H, OC*H*_3_), 5.93 (ddd, ^2^ *J*_*H–P*_ = 13.4 Hz, ^3^ *J*_*H–H*_ = 12.6 Hz, ^4^ *J*_*H–H*_ = 2.6 Hz, 1H, C*H*), 6.49 (dd, ^3^ *J*_*H–H*_ = 8.1 Hz, ^4^ *J*_*H–H*_ = 2.2 Hz, 1H, Ar-*H*), 6.53 (d, ^4^ *J*_*H–H*_ = 2.2 Hz, 1H, Ar-*H*), 7.27 (d, ^3^ *J*_*H–H*_ = 8.1 Hz, 1H, Ar-*H*), 7.32 (dd, ^3^ *J*_*H–P*_ = 40.3 Hz, ^3^ *J*_*H–H*_ = 12.6 Hz, 1H, C*H*), 8.89 (s, 1H, exchange with D_2_O, PN*H*); ^13^C NMR (100 MHz, DMSO-*d*_6_): δ 55.55, 100.89 (d,^3^ *J*_*C–P*_ = 9.3 Hz), 106.68, 111.80 (d,^1^ *J*_*C–P*_ = 141.3 Hz), 113.37, 131.66, 141.46, 143.35, 160.90; ^31^P NMR (162 MHz, DMSO-*d*_6_): δ 7.11; HRMS (*m*/*z*): calcd for C_9_H_9_NO_3_P ([M–H]^−^), 210.0326; found, 210.0321.

### Synthesis of 2,7-Dihydroxybenzo­[e]­[1,2]­oxaphosphinine 2-Oxide (**9**)

BBr_3_ was added to a solution of **7** (50 mg, 1.0 equiv) in dry DCM (6 mL) cooled at 0 °C and the reaction mixture was stirred at r.t. overnight. The suspension was filtered with Celite and the solvent was removed under vacuum. The powder was treated with water, the suspension was filtered and the crude product was purified by flash chromatography (MeOH/DCM 5–10%) to give **9**. Yield 37%; ^1^H NMR (400 MHz, DMSO-*d*_6_): δ 6.03 (dd, ^2^ *J*_*H–P*_ = 19.8 Hz, ^3^ *J*_*H–H*_ = 12.5 Hz, 1H, C*H*), 6.50 (d, ^4^ *J* = 2.3 Hz, 1H, Ar-*H*), 6.58 (dd, ^3^ *J* = 8.2 Hz, ^4^ *J* = 2.3 Hz, 1H, Ar-*H*), 7.24 (d, ^3^ *J* = 8.2 Hz, 1H, Ar-*H*), 7.27 (d, ^3^ *J*_*H–P*_ = 41.9 Hz, ^3^ *J*_*H–H*_ = 12.5 Hz, 1H, C*H*), 10.15 (s, 1H, exchange with D_2_O, O*H*); ^13^C NMR (100 MHz, DMSO-*d*_6_): δ 104.35 (d,^3^ *J*_*C–P*_ = 6.7 Hz), 112.23, 113.83 (d,^1^ *J*_*C–P*_ = 168.4 Hz), 114.40 (d,^2^ *J*_*C–P*_ = 20.2 Hz), 133.03 (d,^4^ *J*_*C–P*_ = 1.1 Hz), 142.07, 153.46 (d,^3^ *J*_*C–P*_ = 7.7 Hz), 160.08 (d,^4^ *J*_*C–P*_ = 1.4 Hz); ^31^P NMR (162 MHz, DMSO-*d*_6_): 6.26; HRMS (*m*/*z*): calcd for C_8_H_6_O_4_P ([M–H]^−^), 197.0009; found, 197.0003.

### Synthesis of 2,7-Dimethoxybenzo­[e]­[1,2]­oxaphosphinine 2-Oxide (**10**)

Derivative **7** (60 mg, 1.0 equiv) was added portion wise to SOCl_2_ cooled at 0 °C and the reaction mixture was stirred at reflux temperature for 4 h. The thionyl chloride was removed under vacuum and residue was treated with dry MeOH and the solution was stirred at r.t for 0.5 h. The solvent was removed under vacuum and the crude product was purified by flash chromatography (MeOH/DCM 5–10%) to give **10**. Yield 69%; ^1^H NMR (400 MHz, DMSO-*d*_6_): δ 3.66 (d, ^3^ *J*_*H–P*_ = 12.3 Hz, 3H, OC*H*_3_), 3.85 (s, 3H, OC*H*_3_), 6.22 (dd, ^2^ *J*_*H–P*_ = 20.2 Hz, ^3^ *J*_*H–H*_ = 12.5 Hz, 1H, C*H*), 6.86 (dd, ^3^ *J* = 8.4 Hz, ^4^ *J* = 2.4 Hz, 1H, Ar-*H*), 6.89 (d, ^4^ *J* = 2.4 Hz, 1H, Ar-*H*), 7.68 (dd, ^3^ *J* = 12.5 Hz, ^4^ *J*_*H–P*_ = 44.4 Hz, 1H, C*H*); ^13^C NMR (100 MHz, DMSO-*d*_6_): δ 53.87 (d,^3^ *J*_*C–P*_ = 6.5 Hz), 56.75, 104.42 (d,^3^ *J*_*C–P*_ = 7.4 Hz), 108.48 (d,^1^ *J*_*C–P*_ = 167.9 Hz), 111.68, 114.53 (d,^2^ *J*_*C–P*_ = 20.2 Hz), 132.27 (d,^4^ *J*_*C–P*_ = 1.3 Hz), 146.13, 153.65 (d,^3^ *J*_*C–P*_ = 8.7 Hz), 162.67 (d,^4^ *J*_*C–P*_ = 1.5 Hz, *C*CHCHP); ^31^P NMR (162 MHz, DMSO-*d*_6_): 10.54; HRMS (*m*/*z*): calcd for C_10_H_12_O_4_P ([M + H]^+^), 227.0468; found, 227.0473.

### Carbonic Anhydrase Inhibition Assay

An Applied Photophysics stopped-flow instrument has been used for assaying the CA-catalyzed CO_2_ hydration activity.^13^ Phenol red (at a concentration of 0.2 mM) has been used as indicator, working at the absorbance maximum of 557 nm, with 20 mM Hepes (pH 7.5) as buffer and 20 mM Na_2_SO_4_ (for maintaining constant the ionic strength), following the initial rates of the CA-catalyzed CO_2_ hydration reaction for a period of 10–100 s. The CO_2_ concentrations ranged from 1.7 to 17 mM for the determination of the kinetic parameters and inhibition constants. For each inhibitor, at least six traces of the initial 5–10% of the reaction have been used for determining the initial velocity. The uncatalyzed rates were determined in the same manner and subtracted from the total observed rates. Stock solutions of inhibitor (10 mM) were prepared in DMSO, and dilutions up to 0.1 nM were done thereafter with the assay buffer. Inhibitor and enzyme solutions were preincubated together at room temperature before assay to allow for the formation of the E-I complex. The inhibition constants were obtained by nonlinear least-squares methods using PRISM 7 and the Cheng-Prusoff equation and represent the mean from at least three different determinations.^15^ The enzyme concentrations were in the range of 5–14 nM. All hCA isoforms were recombinant ones obtained in-house.^15^

#### Molecular Modeling

##### Molecular Docking

Docking simulations were performed with the GOLD software^16^ using the ChemPLP function for both docking and scoring purposes. The crystallographic structure of human hCA IX in complex with a sulfonamide inhibitor coded by PDB-ID 5FL4 ^17^ was used as a rigid receptor in molecular docking simulations. Upon removal of the inhibitor from the crystallographic structure, the Zn-bound water molecule was modeled as described previously.^18^ Ligands were sketched in Picto version 4.4.0.4 (OpenEye Cadence molecular Sciences, Santa Fe, NM) and converted into 3D structures by Omega version 3.1.0.3 (OpenEye Cadence molecular Sciences, Santa Fe, NM). Ligands protonation was verified by QUACPAC version 2.0.0.3 (OpenEye Cadence molecular Sciences, Santa Fe, NM) and by MoKa version 4.0.12 (Molecular Discovery, UK). Molecular docking was carried out in two different configurations of the Zn-bound water molecule (i): the *”on”* mode that force the presence of the water molecule in the receptor’s structure, and (ii) the “*Toggle”* mode that allows the docking function to decide whether the water should be kept or displaced by the ligand during docking.

### MD Simulations

The system was prepared with the AmberTool Leap, the ff14SB and the General Amber Force Field (GAFF) were used for the parametrization of the protein and the ligand, respectively.^19^ To avoid bias from docking simulations, the intermolecular recognition and binding between **7** and hCA IX was simulated by placing the two molecules in a simulation box at a random reciprocal orientation and at a distance higher than 20 Å.^20^ The crystallographic structure of hCA IX coded by PDB: 5FL4 was used as a receptor in the intermolecular recognition MD simulation. A rectilinear box of TIP3P type water molecules buffering 6 Å from the molecules was used, Na^+^ ions were added in the simulation box up to system charge neutrality. MD simulations were run with AMBER22, using a time step of 2 ps.^21^ According to a consolidated MD protocol,^22,23^ the solvent was first energy minimized for 500 steps with the steepest descent algorithm (SD) followed by 2500 steps with the conjugate gradient algorithm (CG) while keeping the solute frozen. Then, the solvated solute was energy minimized for 1000 steps with the SD and subsequent 5000 steps with the CG before being heated to 300 K at constant volume with the Langevin thermostat for 1 ns. System density was then equilibrated with the Berendsen barostat at constant pressure for 1 ns before 50 ns of MD simulations were produced at constant pressure. Finally, 2.5 μs of unrestrained MD trajectories were produced for analysis purposes. Analysis of MD trajectories was carried out with CPPTRAJ.^24^

### DFT-QM/MM Calculations

QM/MM calculations were carried out using Gaussian16 ONIOM method.^25,26^ The starting point for these calculations was the hCA IX/**7** complex obtained from MD simulations. The system was divided into two different layers, i.e., a High Layer (HL) and a Low Layer (LL) which were treated at the QM level of theory (DFT) and the MM level of theory, respectively. The TAO toolkit was used to generate the input files for the optimization of **7**. The B3LYP functional was used, with the 3-21g* basis set. The HL is composed of **7**, the catalytic Zn­(II) ion, the Zn-bound OH^–^, the imidazole ring of Zn-binding histidine residues (His94, His96, and His119) and by the Thr199, whereas the remaining part of the system was computed at the MM level of theory. To maintain consistency with the sulfocoumarin study,^6^ the same HL was selected. Accordingly, the proton of Zinc coordinate water molecule oriented furthest from **7** was deleted. The cut between layers was operated at the α-carbon atoms.

#### Crystallographic Studies

hCA II protein was prepared as previously described.^27^ Crystals of hCA II in complex with **7** were obtained by soaking preformed enzyme crystals in a solution containing 50 mM inhibitor in the crystallization buffer (1.3 M sodium citrate and 0.1 M Tris-HCl, pH 8.0). Before data collection, crystals were flash-cooled in liquid nitrogen using reservoir solution supplemented with 20% (v/v) glycerol as a cryoprotectant. A complete data set was collected at 100 K at the Synchrotron source Elettra in Trieste, Italy, using the Dectris Pilatus 6M detector. Intensity data were processed and scaled using the program HKL3000.^28^ Crystal parameters and data processing statistics are summarized in Table S2. The structure of the complex was analyzed by different Fourier techniques, using hCA II crystallized in the P2_1_ space group (PDB 6EQU)^29^ as the starting model after removal of nonprotein atoms. Positional and individual B-factor refinement were performed using REFMAC5.^30,31^ Restraints for inhibitor bond angles and distances were taken from the Cambridge Structural Database (Groom et al. 2016), while standard restraints were applied to protein bond angles and distances throughout refinement. Water molecules were built into peaks >3σ in |Fo| – |Fc| maps that demonstrated appropriate hydrogen-bonding geometry. The model was refined to final crystallographic *R*_work_ and *R*_free_ values of 0.152/0.172 in the 41.2–1.65 Å resolution range (Table S2). Refinement statistics are summarized in Table 1. Coordinates and structure factors have been deposited with the Protein Data Bank (accession code: 9TYE).

#### NMR Study

Kinetic time-course experiments were conducted at different incubation times (*t* = 0, 1, 2, 3, 4, 5, and 6 h) following enzyme–inhibitor complex formation at 25 °C. Inhibitor stock solutions (10^–1^ M in DMSO-*d*_6_) were diluted into enzyme solutions (hCA at 10^–5^ M in 20 mM HEPES buffer, pH 7.4) to achieve an inhibitor concentration of 1 × 10^–3^ M. For NMR analysis, 30 μL of the enzyme–inhibitor mixture was further diluted with 270 μL of DMSO-*d*_6_ to a final inhibitor concentration of 10^–4^ M. ^31^P NMR spectra were recorded on a Bruker 400 MHz Avance III spectrometer equipped with a Bruker PBBO probe at 25 °C in 5 mm NMR tubes. Spectra were acquired with 128 scans, 2.5 s relaxation delay, and processed using TopSpin 4.X software.

#### MS Study

HPLC–MS, HPLC–MS/MS, and HPLC–HRMS analyses were performed to confirm the elemental composition and support the structural identification of the compound formed after 6 h incubation of compound **10** in a solution containing carbonic anhydrase IX (hCA IX). Chromatographic separations were carried out using the HPLC system described above, equipped with a Poroshell 120 EC-C18 column (2.1 × 150 mm, 2.7 μm particle size; Agilent, USA). The mobile phase consisted of 10 mM formic acid and 5 mM ammonium formate in Milli-Q water (solvent A) and methanol (solvent B). The elution program started at 95% solvent A, which was linearly decreased to 5% over 9 min and held for 10 min. The initial conditions (95% solvent A) were then restored within 1 min, followed by a column re-equilibration step of 10 min, resulting in a total run time of 30 min. The mobile phase flow rate was set at 0.20 mL min^–1^, the injection volume was 5 μL, and the column temperature was maintained at 40 °C. MS analyses were performed in positive ion mode, acquiring full-scan chromatographic profiles over an *m*/*z* range of 150–600. HRMS experiments were carried out over the same *m*/*z* range, setting the instrument resolution to 30,000 fwhm in order to ensure an adequate data acquisition rate. MS/MS experiments were conducted by isolating the protonated molecular ion ([M + H]^+^ = 245 *m*/*z*) of the unknown compound. Fragmentation was induced using an excitation amplitude of 25 au applied for 50 ms, and product ion spectra were recorded in the *m*/*z* range 80–270.

#### Antitumor Study

The four cancer cell lines (MCF-7, MDAMB-231, HCT116, and PC-3) were obtained from the American Type Culture Collection (ATCC). The cells were propagated in DMEM supplemented with 10% heat-inactivated fetal bovine serum, 1% l-glutamine (2.5 mM), HEPES buffer (10 mM) and gentamicin (50 μg/mL). All cells were maintained at 37 °C in a humidified atmosphere with CO_2_ (5%). Cytotoxicity was then evaluated using the MTT assay,^32^ as previously reported.^33^ For the ELISA Immunoassay, the levels of the apoptotic markers (Bax, caspase-3, caspase-9, cytochrome C, and p53), as well as the antiapoptotic marker (Bcl-2), were evaluated using colorimetric ELISA kits according to the manufacturer’s instructions, as previously reported.^34^

## Supplementary Material







## Data Availability

Authors will release the atomic coordinates upon article publication.

## Notes

Crystal structure PDB ID 9TYE (CIF)
A separate molecular formula strings file for biologically tested compounds (CSV)
Figures and Tables, NMR spectra, HPLC traces (PDF)

## Abbreviations

CA: carbonic anhydrase
DOX: doxorubicin
hCA: human carbonic anhydrase
IC_50_: half maximal inhibitory concentration
K_I_: inhibition constant
MD: molecular dynamics
MS: mass spectrometry
MTT: 3-(4,5-dimethylthiazol-2-yl)-2,5-diphenyltetrazolium bromide
NMR: nuclear magnetic resonance
QM/MM: quantum mechanics/molecular mechanics
STA: staurosporine
ZBG: zinc-binding group
