# Supplementary material for: Discovery of a Mixed and Prodrug-Like Inhibition Mechanism for Phosphocoumarins and Phosphoquinolinones against Human Carbonic Anhydrases
Source: J Med Chem. 2026 Apr 29;69(9):11638–48. doi: 10.1021/acs.jmedchem.6c00915 (PMC13181790; doi:10.1021/acs.jmedchem.6c00915)
Supplement: Supplementary file 3 [file jm6c00915_si_003.pdf]

SUPPORTING INFORMATION

Discovery of a Mixed and Prodrug-Like Inhibition Mechanism for Phosphocoumarins and Phosphoquinolinones against Human Carbonic Anhydrases

Alessio Nocentini,[1,†,*] Simone Giovannuzzi,[1,†] Vincenzo Alterio,[2] Alessandro Bonardi,[1] Rudolfs Barons,[3,4] Raivis Zalubovskis,[3,4] Wagdy M. Eldehna,[5] Rossella Aronne,[6] Davide Esposito,[2] Enrico Luchinat,[7,8] Giuseppina De Simone,[2] Gianluca Bartolucci,[1] Paola Gratteri,[1] Mattia Mori,[5,*] Claudiu T. Supuran[1]

[1] NEUROFARBA Department, Section of Pharmaceutical Sciences, University of Florence, 50019 Sesto Fiorentino, Italy
[2] Institute of Biostructures and Bioimaging, National Research Council, 80145 Napoli, Italy.
[3] Latvian Institute of Organic Synthesis, LC-1006, Riga, Latvia
[4] Institute of Chemistry and Chemical Technology, Riga Technical University, LV-1048 Riga, Latvia.
[5] Department of Pharmaceutical Chemistry, Faculty of Pharmacy, Kafrelsheikh University, 33516 Kafrelsheikh, Egypt.
[6] Department of Biotechnology, Chemistry and Pharmacy, University of Siena, 53100 Siena, Italy.
[7] CERM - Magnetic Resonance Center, University of Florence, 50019 Sesto Fiorentino, Italy
[8] Department of Chemistry "Ugo Schiff", University of Florence, 50019 Sesto Fiorentino, Italy

Corresponding author: alessio.nocentini@unifi.it (A.N.); mattia.mori@unisi.it (M.M.)

Supplemental tables and figures **S2**

NMR Spectra **S6**

HPLC traces **S13**

Table S1. Comparison of the docking score of **7** to hCA IX as calculated with two different options to manage the Zn-binding water molecule.

Water molecule option	***ChemPLP Fitness score***
“on”	50.86
“Toggle”	64.58

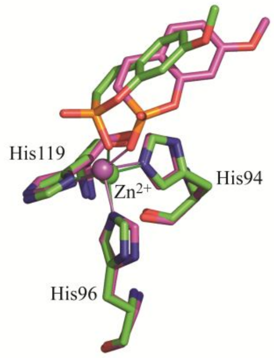

Figure S1. Structural superposition between the X-ray crystallographic pose (green) of compound **7** in hCA II and the pose extracted from MD simulations (magenta) with hCA IX. Compound **7** and the histidine residues are shown as sticks, while the catalytic Zn(II) ion is shown as a sphere. Solid lines indicate zinc ion coordination.

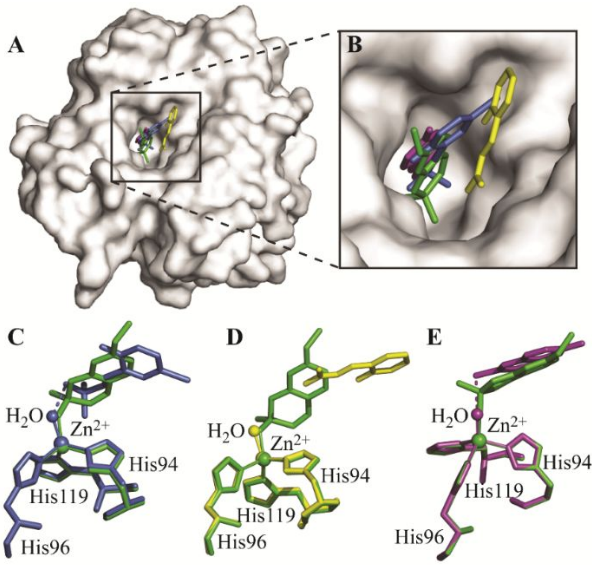

Figure S2. (A) Structural superposition of **7** (green) with hydrolized sulfocoumarin (blue, PDB 4BCW), hydrolized coumarin (yellow, PDB 5BNL), and thioxocoumarin (magenta, PDB 4WL4) bound to the hCA II active site. The protein surface of hCA II/**7** complex is shown. (B) Enlarged view of the active site region. (C) Close-up view of the superposition between **7** and sulfocoumarin. (D) Close-up view of the superposition between **7** and coumarin. (E) Close-up view of the superposition between **7** and thioxocoumarin. In panels C–E, the zinc ion, the three coordinating histidine residues, and, when present, the ZBW molecule are also displayed.

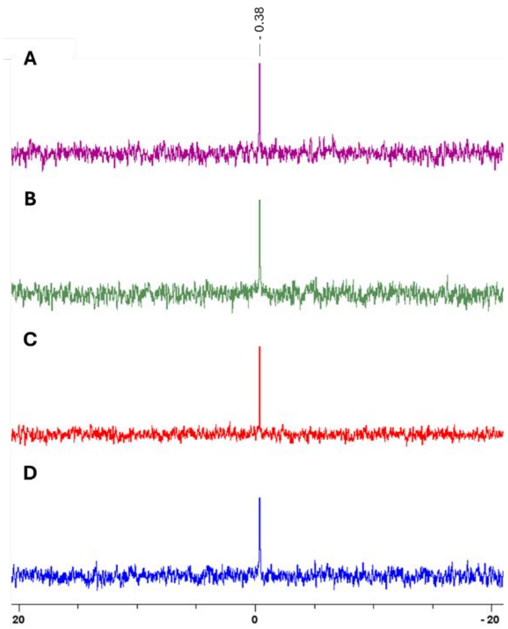

Figure S3. ^{31}P NMR spectra of compound **7** after a 6 h incubation with hCAs A) I, B) II, C) IX, and D) XII. The phosphorus resonance corresponds to the intact acidic phosphocoumarin.

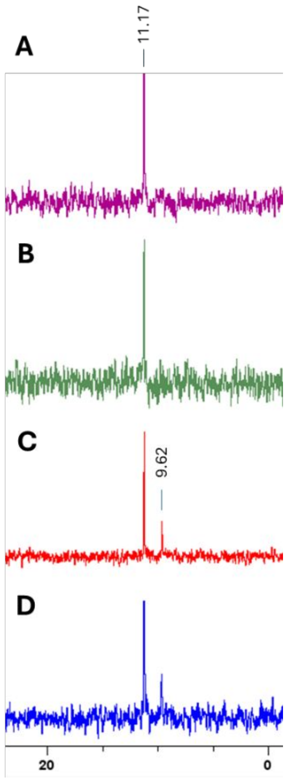

Figure S4. ^{31}P NMR spectra of compound **10** after a 1 h incubation with hCAs A) I, B) II, C) IX, and D) XII. The original phosphorus resonance corresponding to the intact methyl ester phosphocoumarin is shown alongside the new resonance peak indicative of the hydrolyzed phosphoester species.

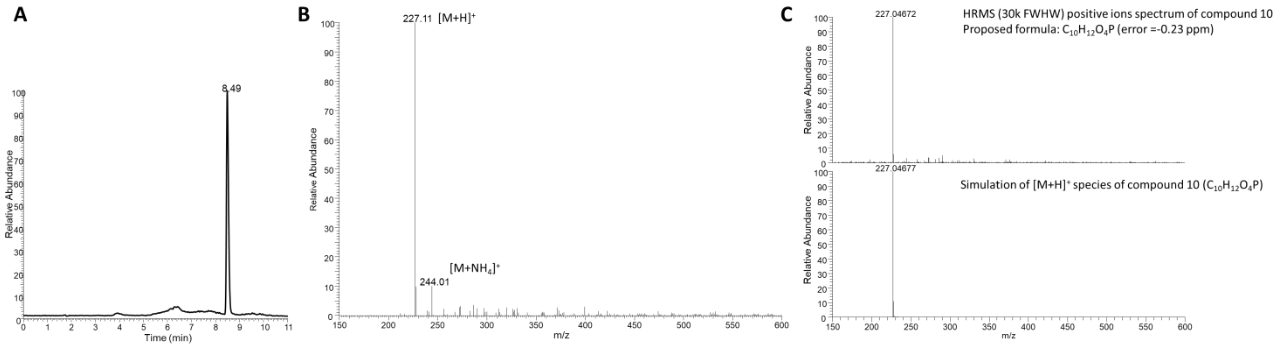

Figure S5. A) HPLC-MS profile of compound **10**; B) MS spectrum positive ions of peak at RT = 5.40 min; C) Comparison between HRMS (30k FWHW) positive ions spectrum of peak at RT = 5.40 min and the simulation of the proposed formula.

Table S2. Data collection and refinement statistics for the hCA **II**/**7** complex. Values in parentheses refer to the highest resolution shell (1.68-1.65 Å).

Crystal parameters	
Space group	$P2_1$
a (Å)	42.5
b (Å)	41.5
c (Å)	72.0
β (°)	104.2
Data collection statistics	
Resolution (Å)	41.2-1.65
Temperature (K)	100
Total reflections	182917
Unique reflections	29354
Completeness (%)	100 (100)
$\langle I \rangle / \langle \sigma(I) \rangle$	14.8 (2.6)
Redundancy (%)	6.2 (6.1)
R_{merge}[a]	0.111 (0.697)
R_{meas}[a]	0.121 (0.760)
R_{pim}[a]	0.048 (0.300)
CC1/2[b]	0.999 (0.759)
Refinement statistics	
Resolution (Å)	41.2-1.65
R_{work}[c] (%)	15.2
R_{free}[c] (%)	17.2
r.m.s.d. from ideal geometry:	
Bond lengths (Å)	0.011
Bond angles (°)	1.7
Number of protein atoms	2039
Number of inhibitor atoms	14
Number of water molecules	97
Average B factor ($Å^2$)	
All atoms	20.1
Protein atoms	19.6
Inhibitor atoms	38.0
Water molecules	26.9

[a] $R_{merge} = \Sigma_{hkl}\Sigma_i|I_i(hkl)-\langle I(hkl)\rangle|/\Sigma_{hkl}\Sigma_i I_i(hkl)$; $R_{meas} = \Sigma_{hkl}\{n(hkl)/[n(hkl)-1]\}^{1/2}\Sigma_i|I_i(hkl)-\langle I(hkl)\rangle|/\Sigma_{hkl}\Sigma_i I_i(hkl)$; $R_{pim} = \Sigma_{hkl}\{1/[n(hkl)-1]\}^{1/2}\Sigma_i|I_i(hkl)-\langle I(hkl)\rangle|/\Sigma_{hkl}\Sigma_i I_i(hkl)$, where $I_i(hkl)$ is the intensity of an observation and $\langle I(hkl)\rangle$ is the mean value for its unique reflection; summations are over all "n" reflections.

[b] $CC1/2 = [\Sigma_i(a_i-\langle a\rangle)/\Sigma_i(b_i-\langle b\rangle)]/[\Sigma_i(a_i-\langle a\rangle)^2\ \Sigma_i(b_i-\langle b\rangle)^2]^{1/2}$; where a_i and b_i are the intensities of unique reflections merged across the observations randomly assigned to subsets A and B, respectively, and $\langle a\rangle$ and $\langle b\rangle$ are their averages.

[c] $R_{factor} = \Sigma_h||F_o(h)|-|F_c(h)||/\Sigma_h|F_o(h)|$, where F_o and F_c are the observed and calculated structure-factor amplitudes, respectively. R_{free} was calculated with 6% of the data excluded from the refinement.

NMR SPECTRA

^{1}H NMR Spectrum of **7** (400 MHz, DMSO-d_6)

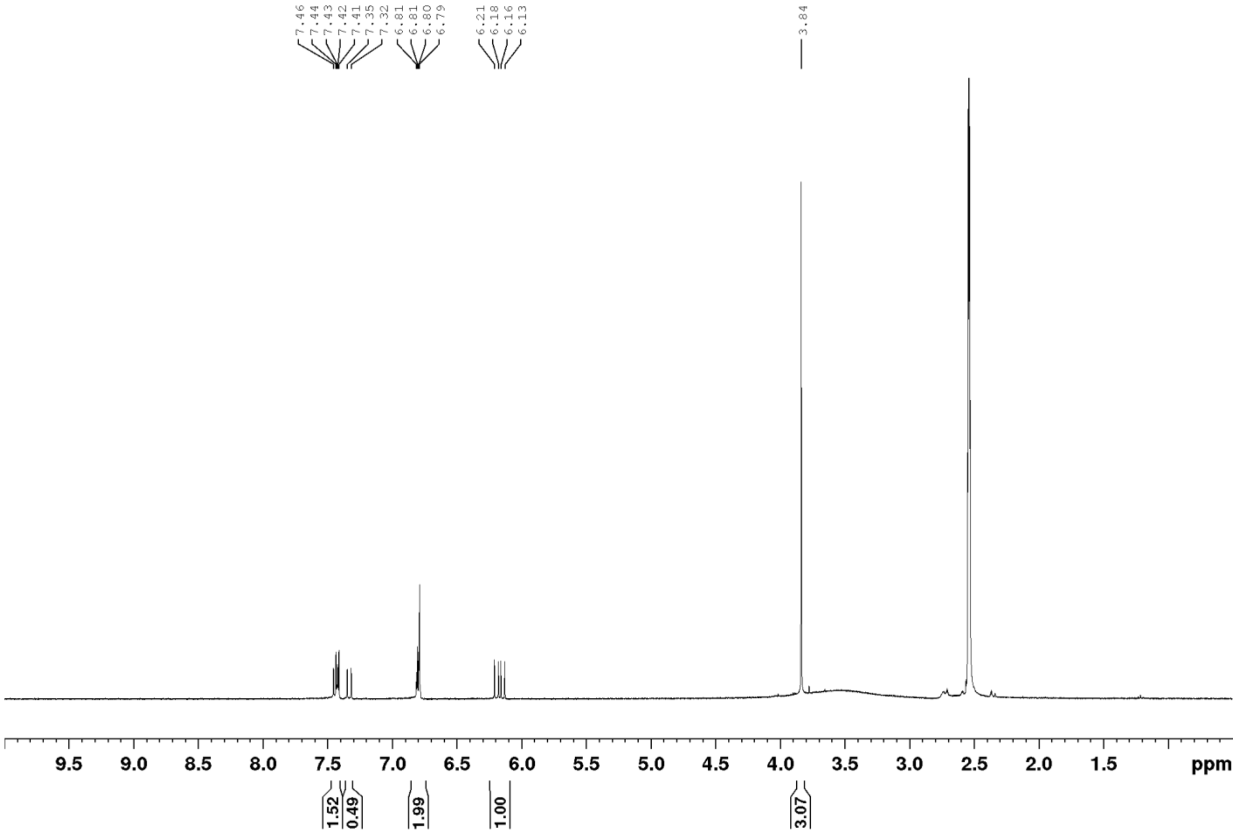

^{13}C NMR Spectrum of **7** (100 MHz, DMSO-d_6)

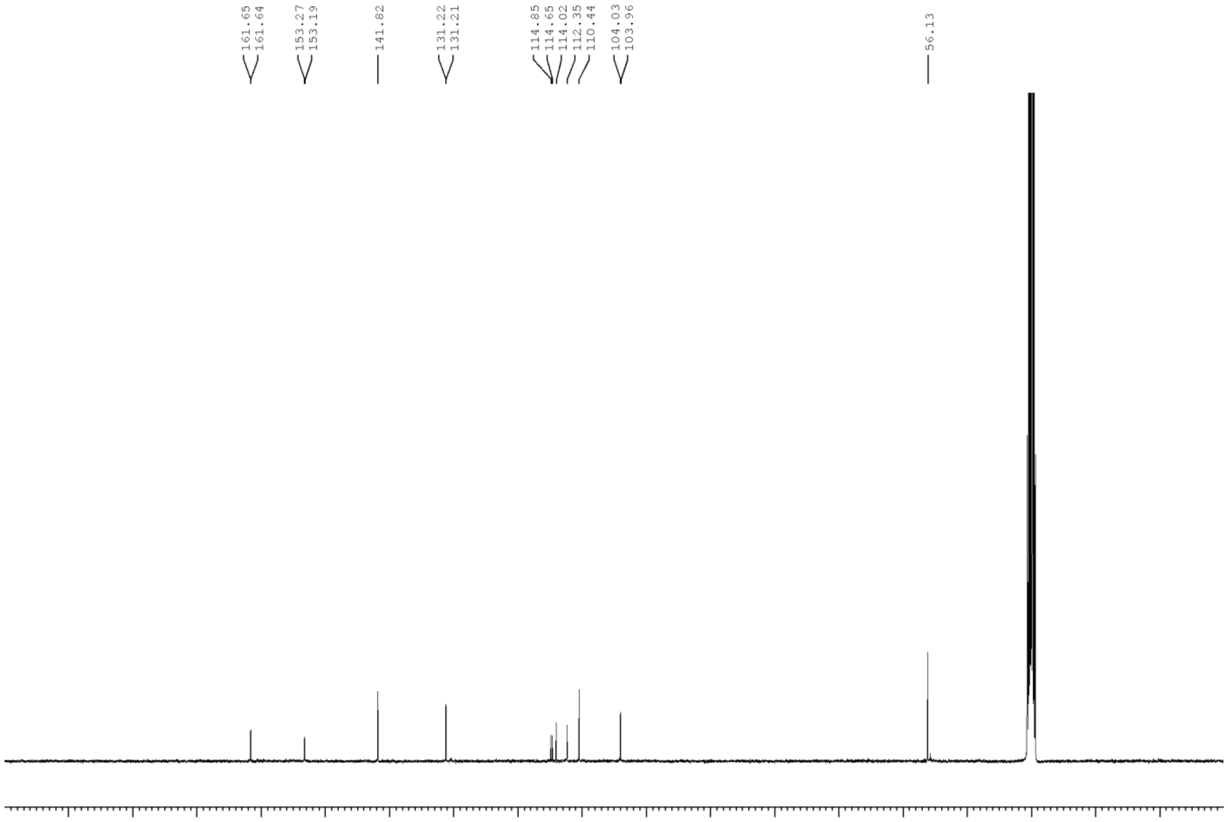

^{31}P NMR Spectrum of **7** (162 MHz, DMSO-d_6)

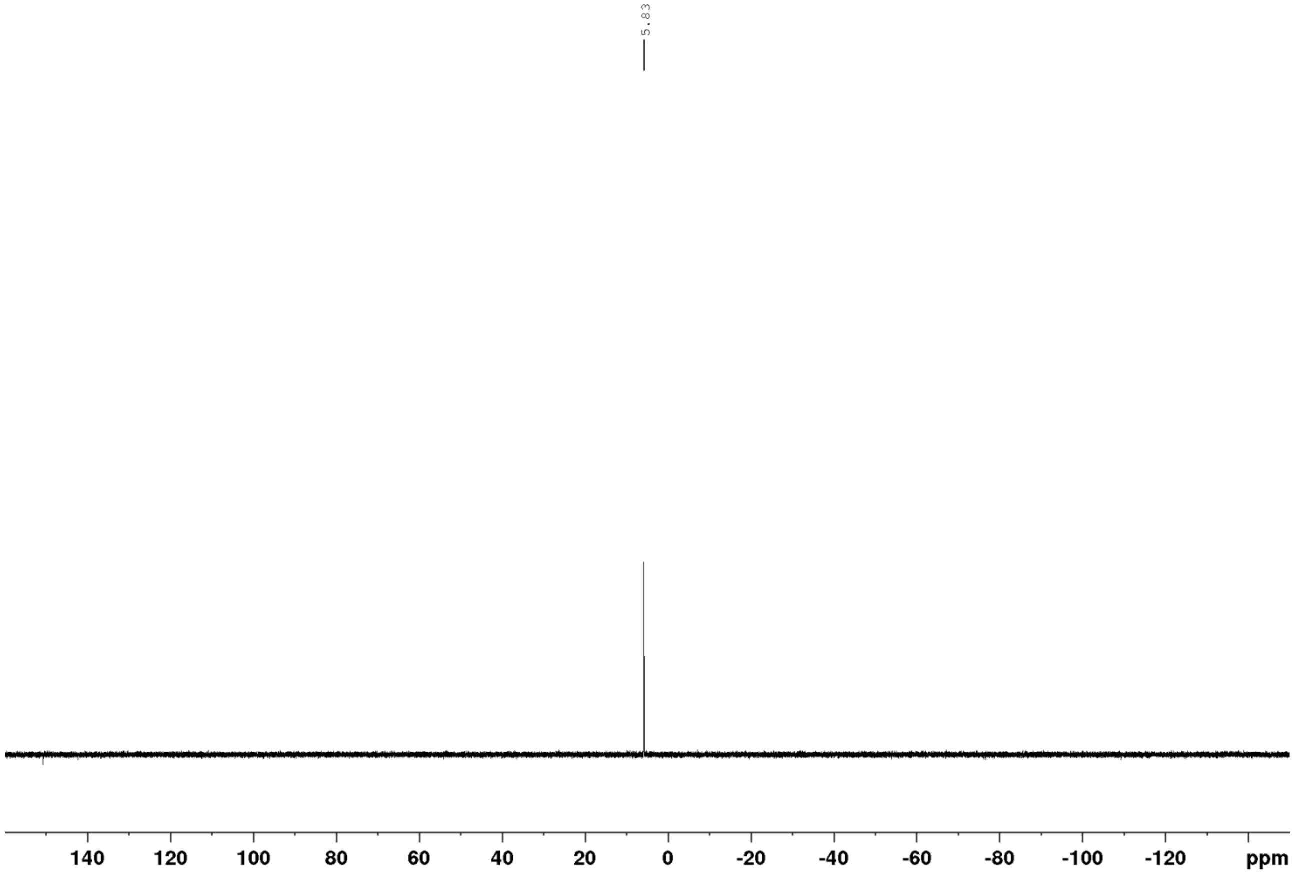

^{1}H NMR Spectrum of **8** (400 MHz, DMSO-d_6)

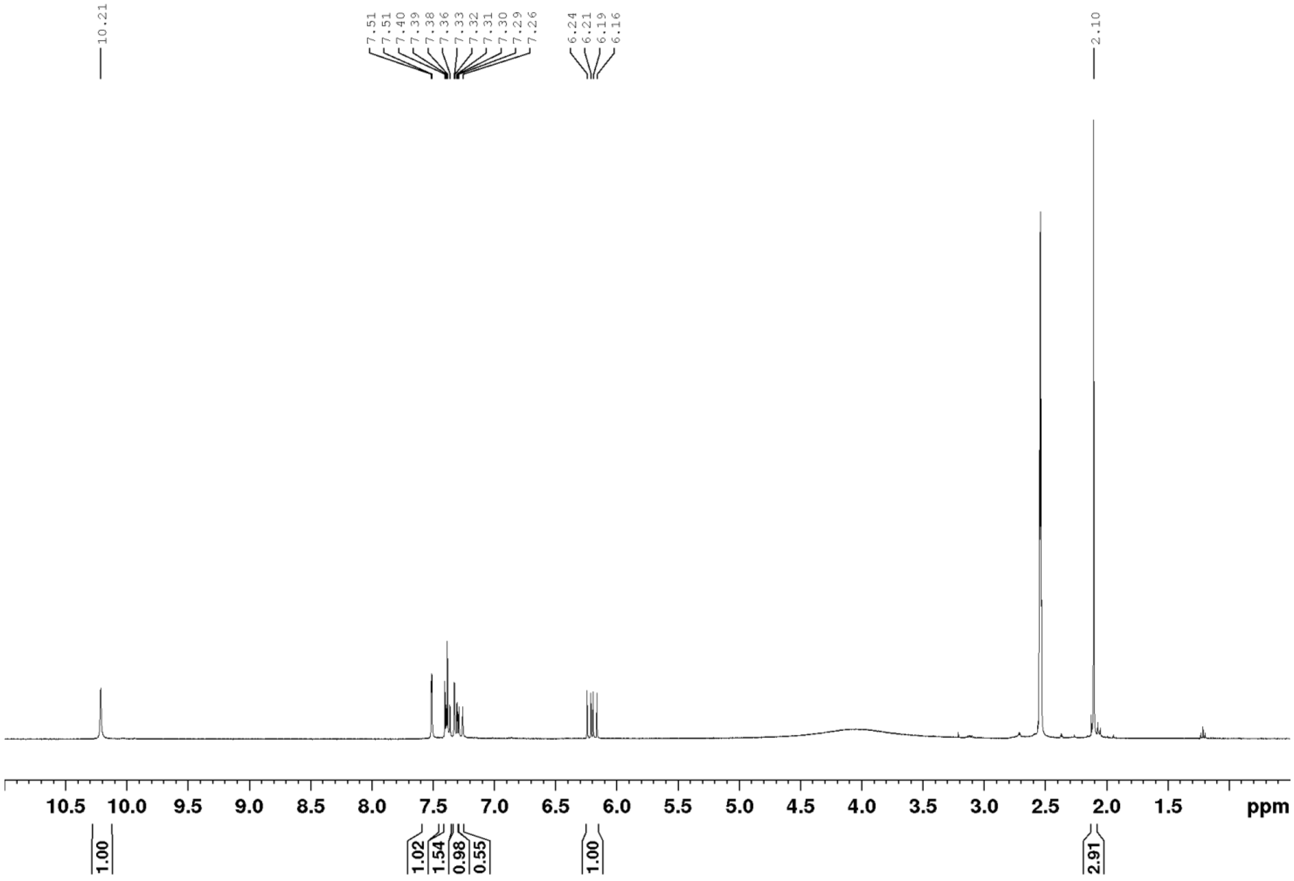

^{13}C NMR Spectrum of **8** (100 MHz, DMSO-d_6)

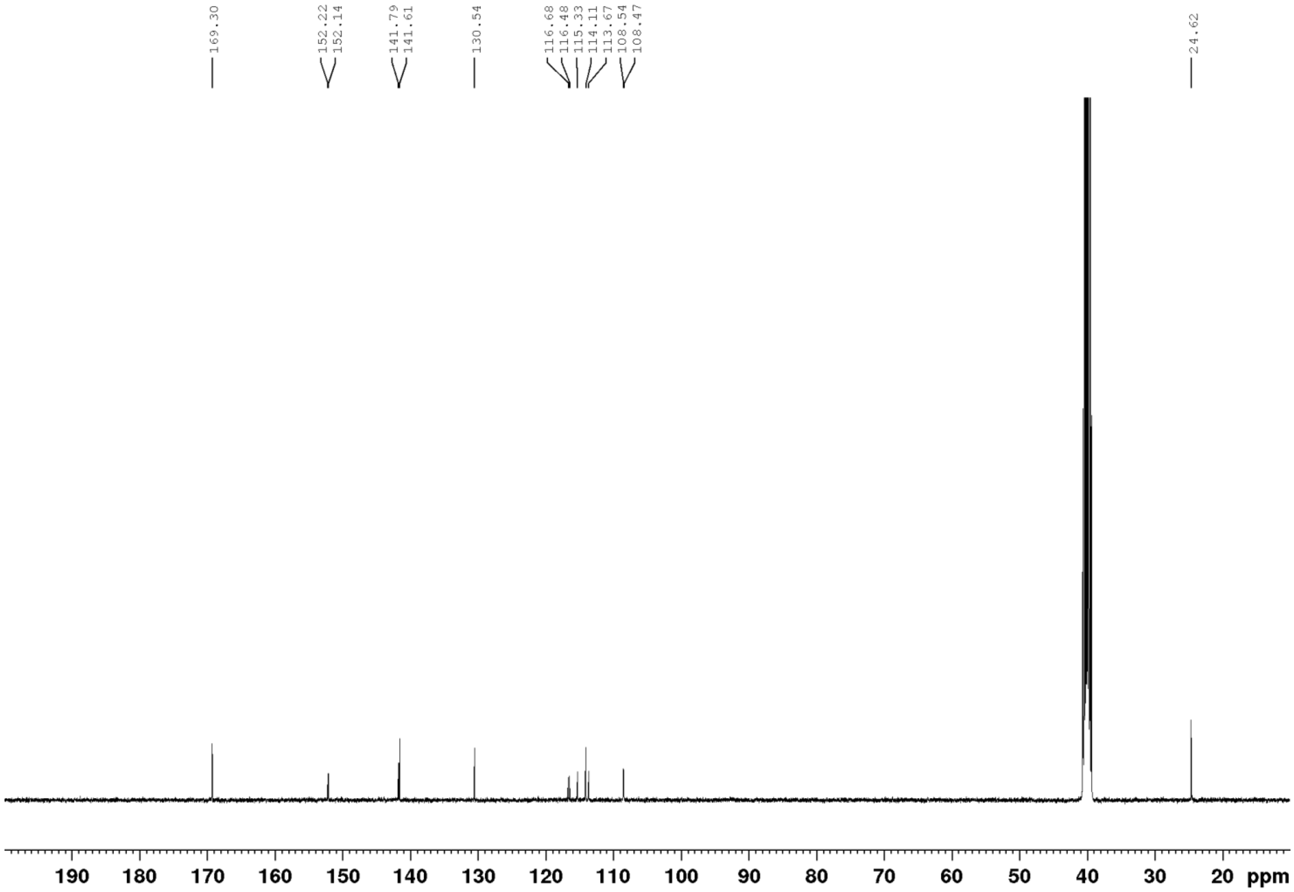

^{31}P NMR Spectrum of **8** (162 MHz, DMSO-d_6)

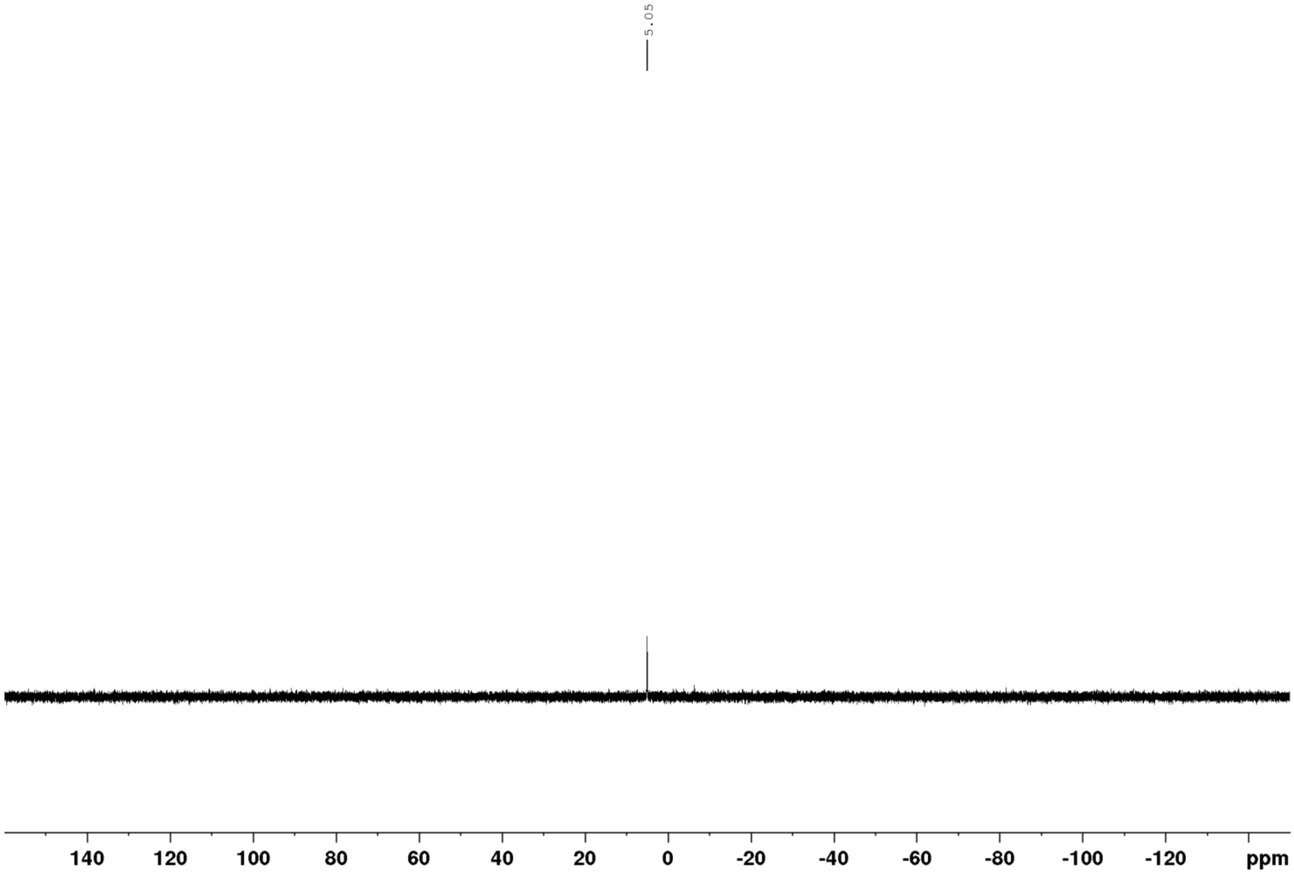

^{1}H NMR Spectrum of **9** (400 MHz, DMSO-d_6)

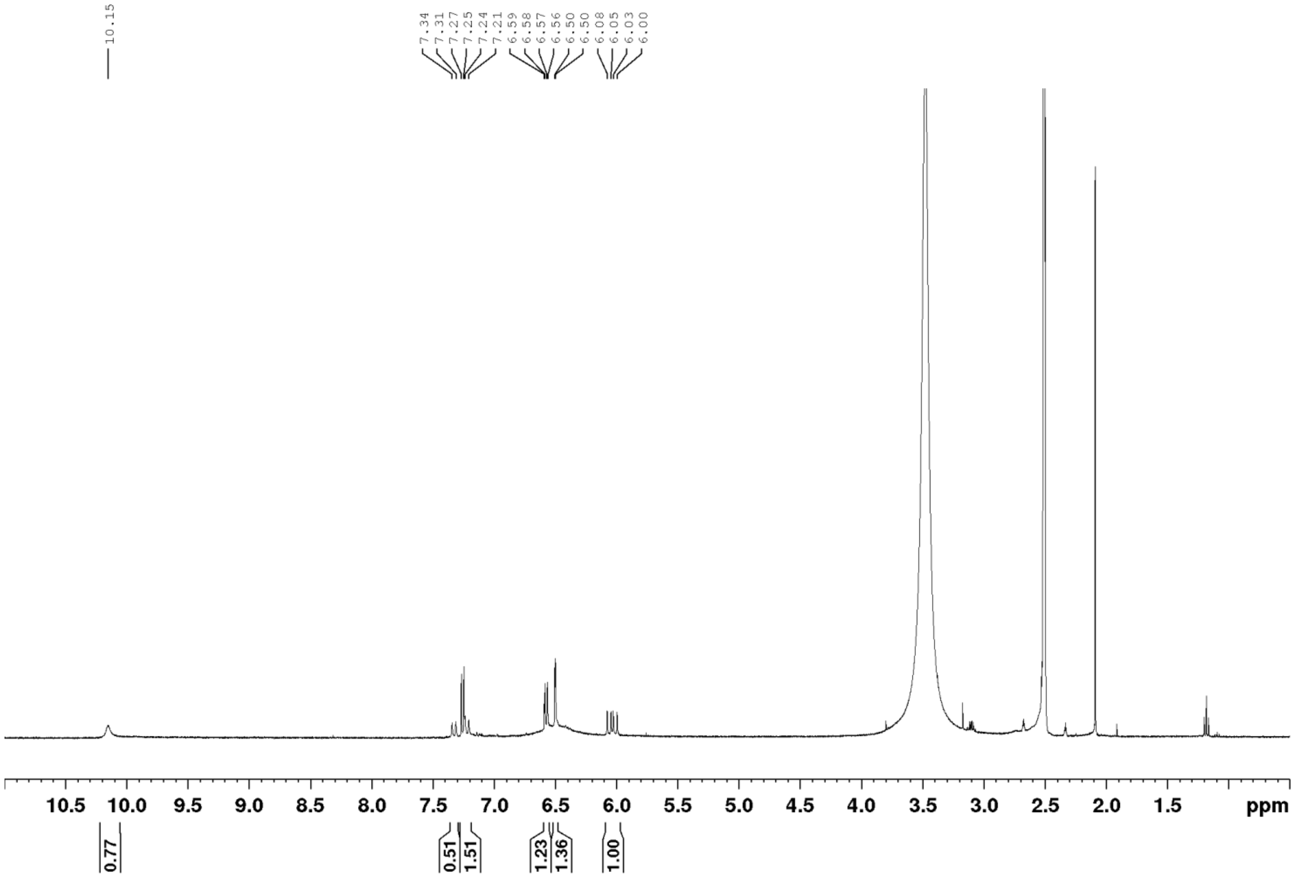

^{31}P NMR Spectrum of **9** (162 MHz, DMSO-d_6)

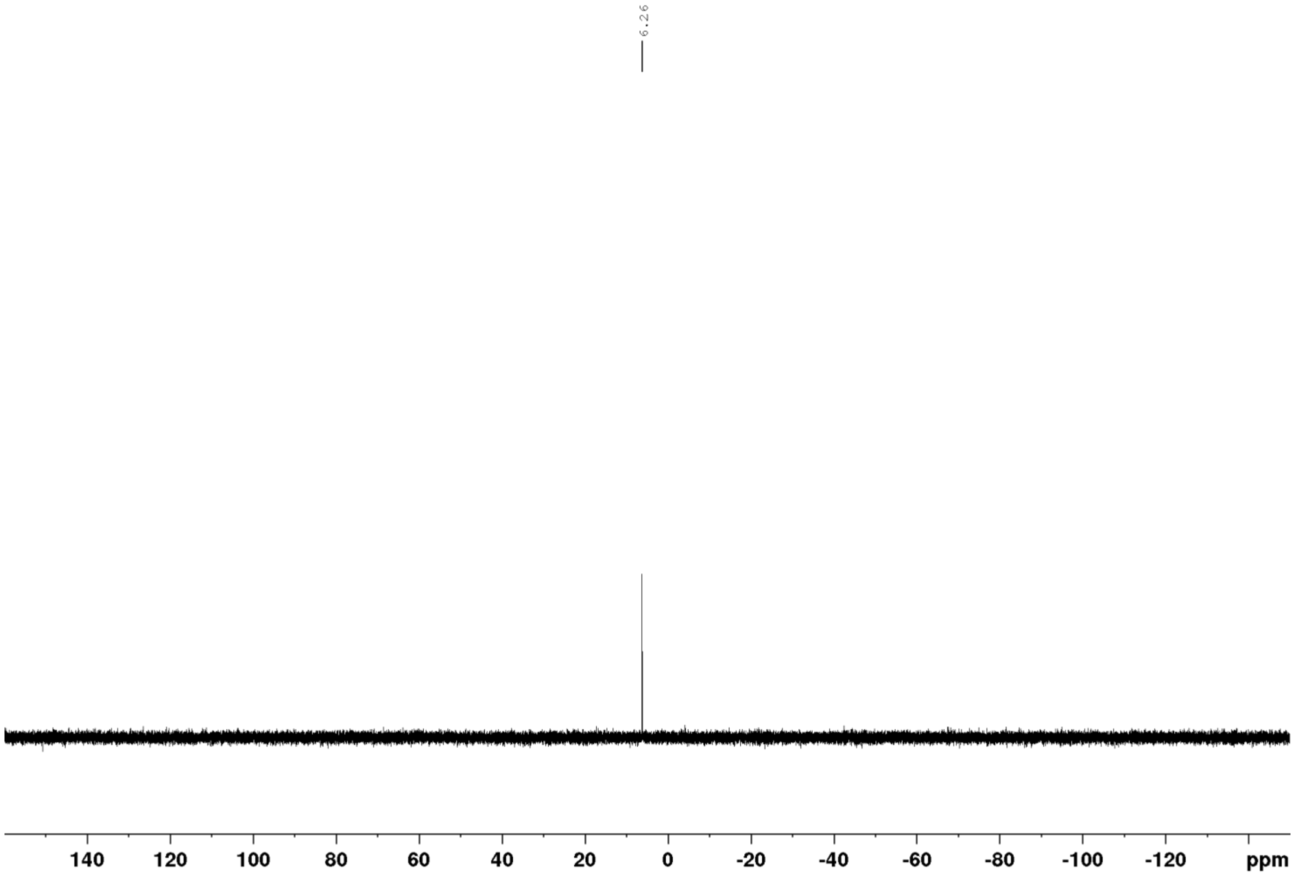

^{1}H NMR Spectrum of **10** (400 MHz, DMSO-d_6)

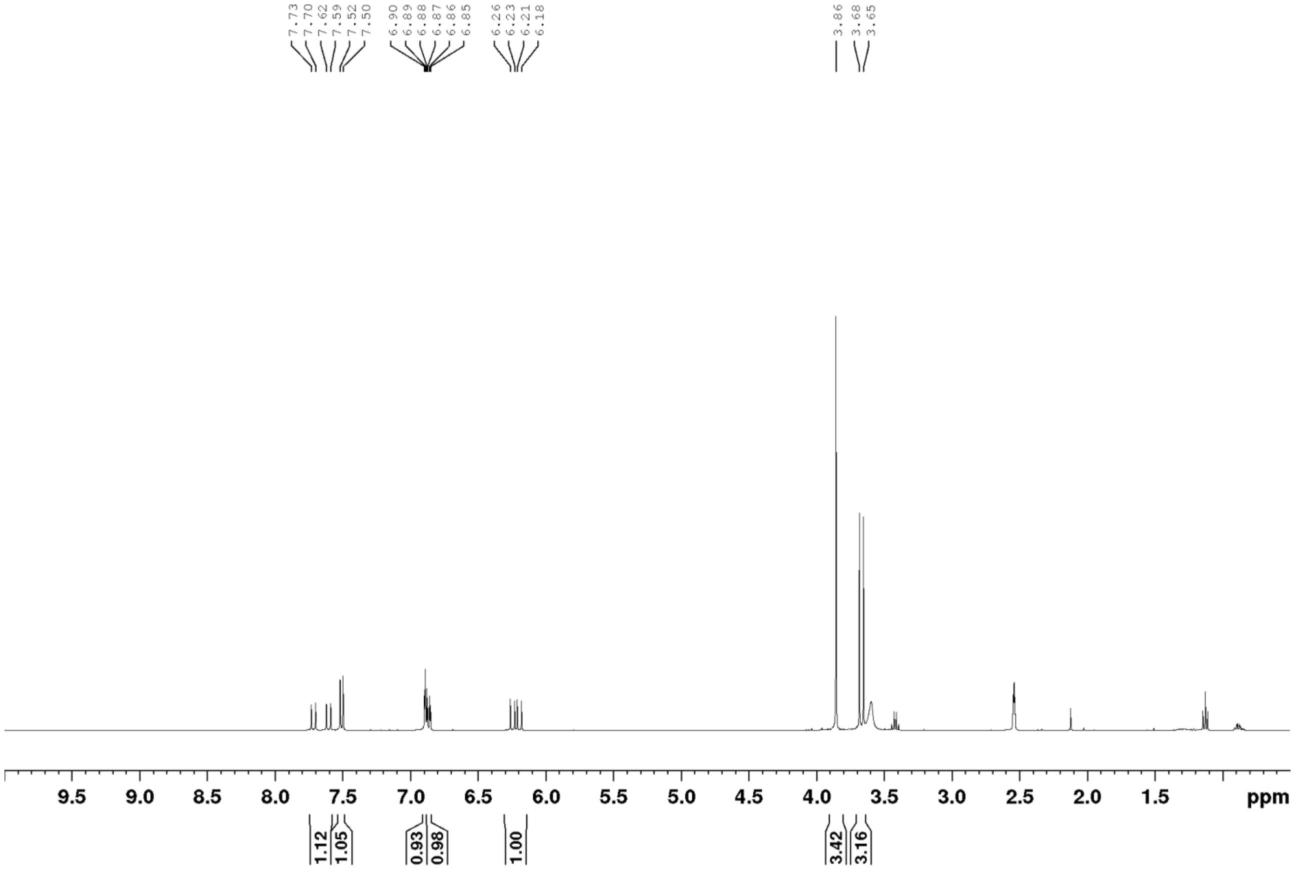

^{13}C NMR Spectrum of **10** (100 MHz, DMSO-d_6)

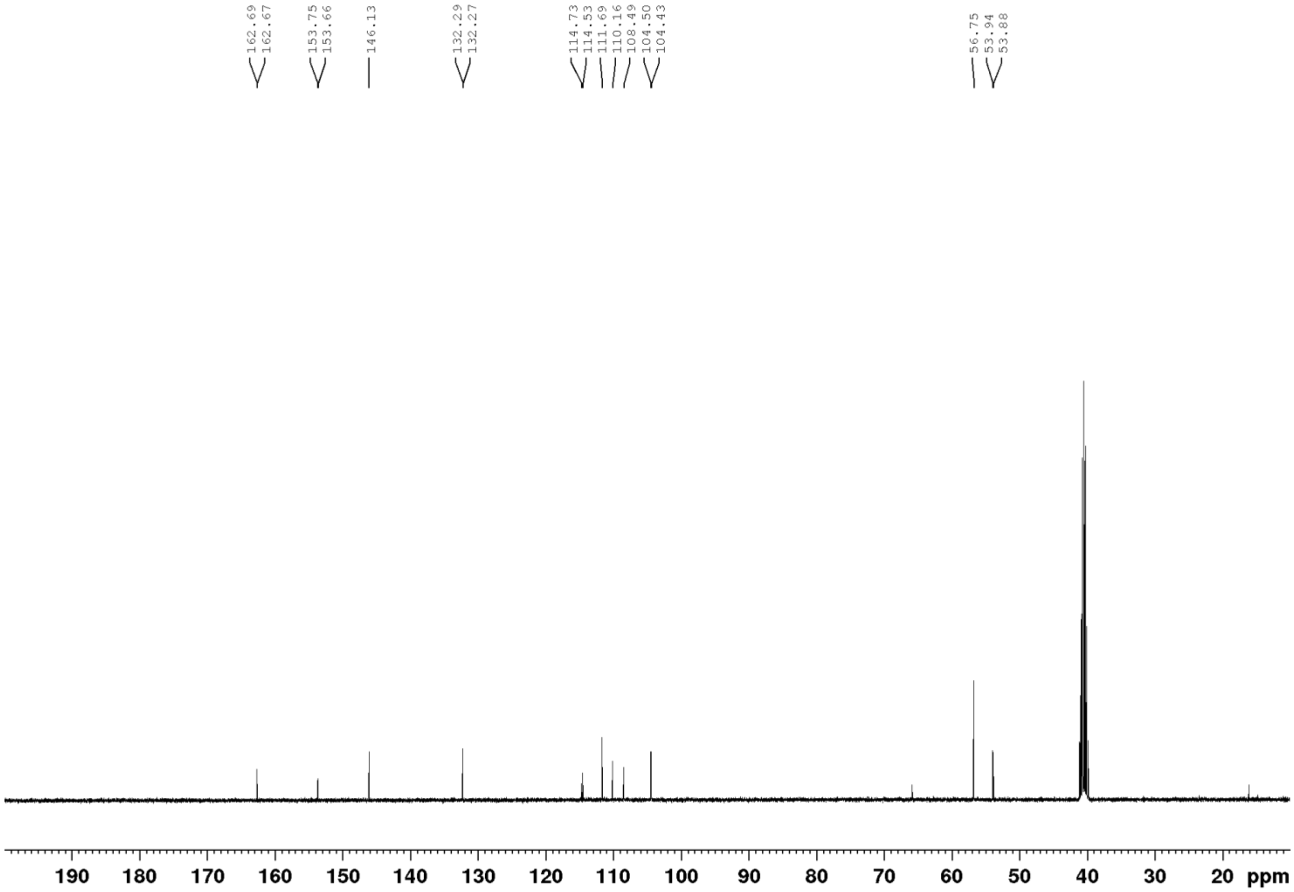

^{31}P NMR Spectrum of **10** (162 MHz, DMSO-d_6)

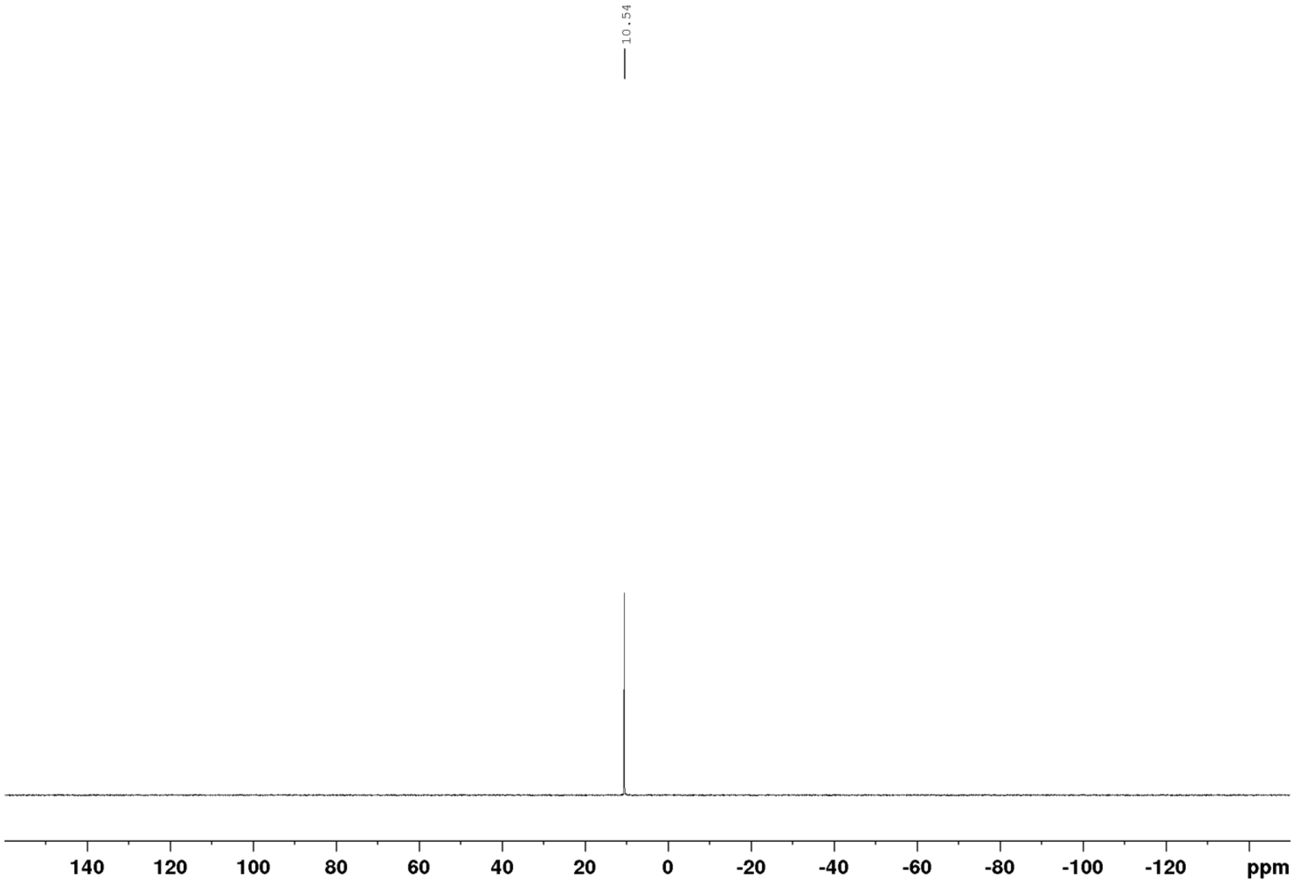

^{1}H NMR Spectrum of **13** (400 MHz, DMSO-d_6)

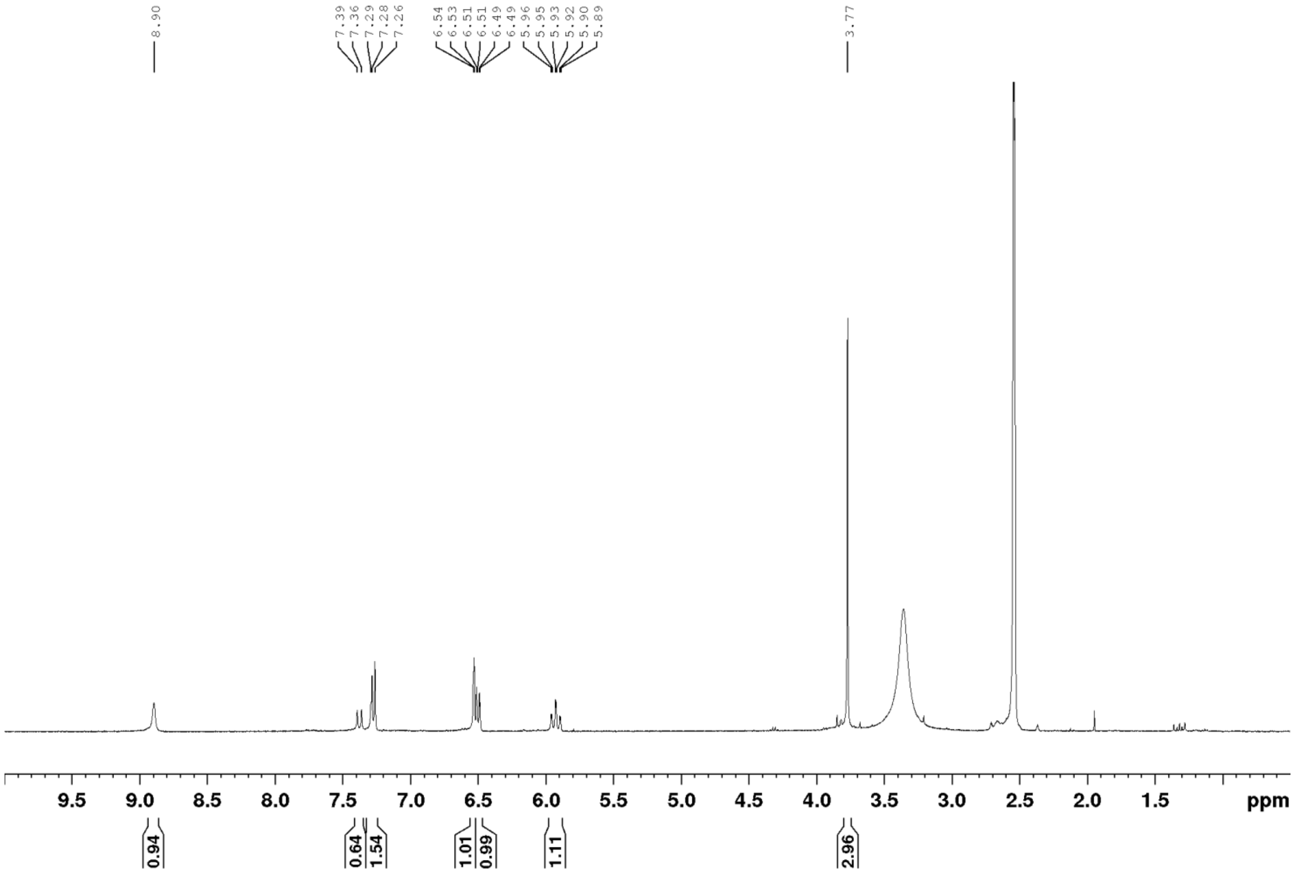

^{13}C NMR Spectrum of **13** (100 MHz, DMSO-d_6)

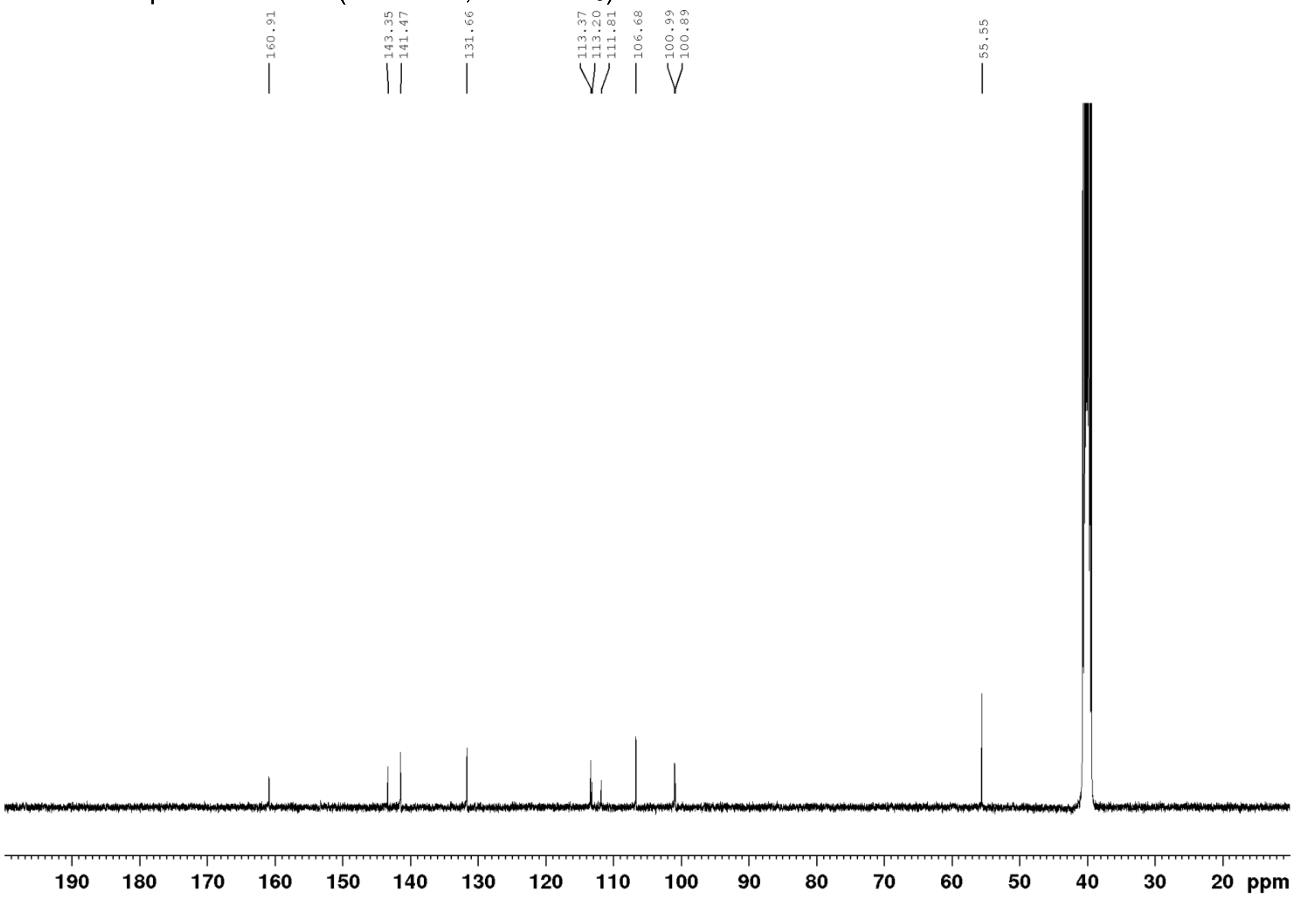

^{31}P NMR Spectrum of **13** (162 MHz, DMSO-d_6)

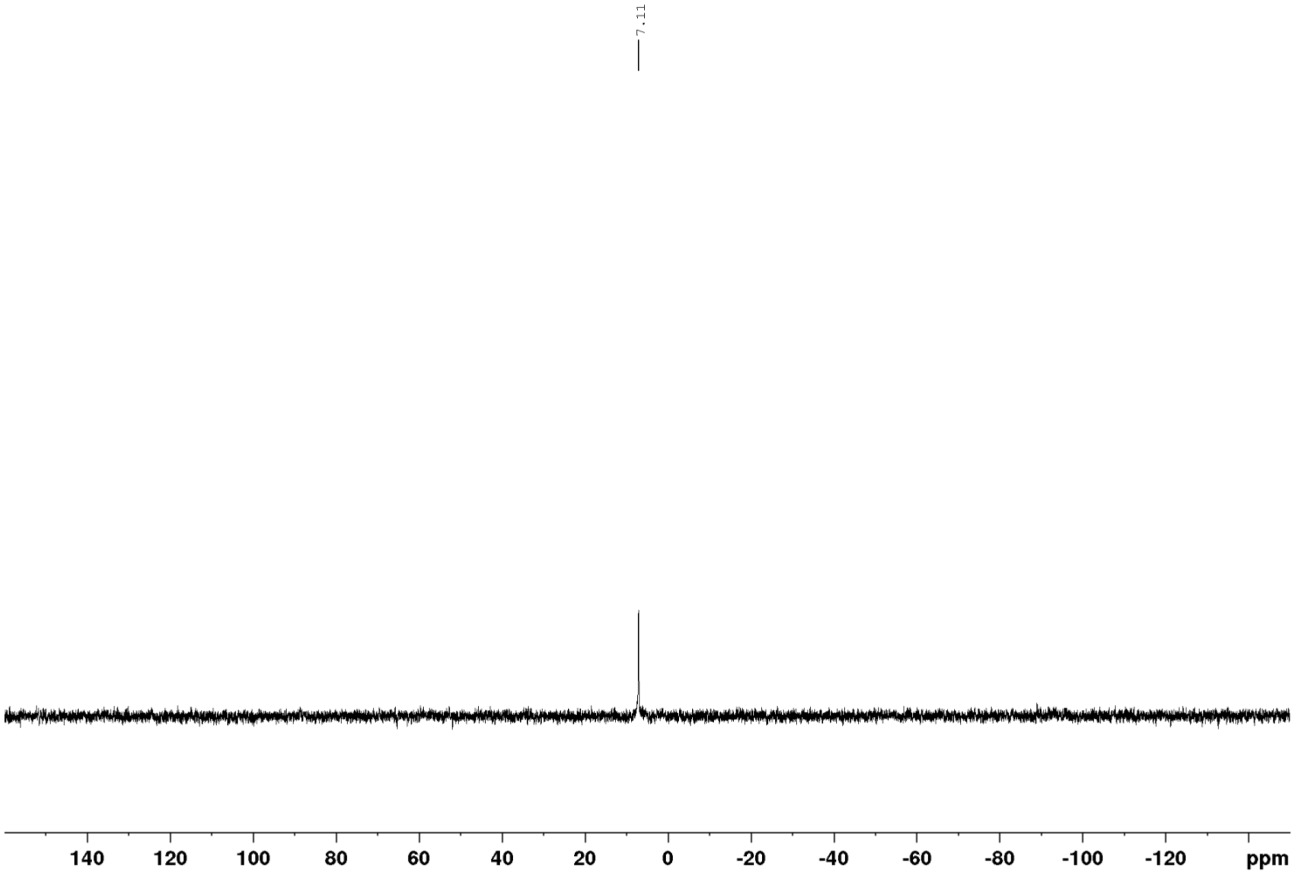

HPLC traces

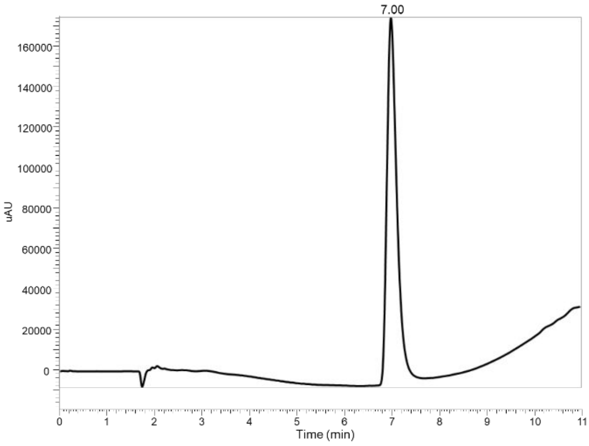

HPLC-UV profile of compound **7.**

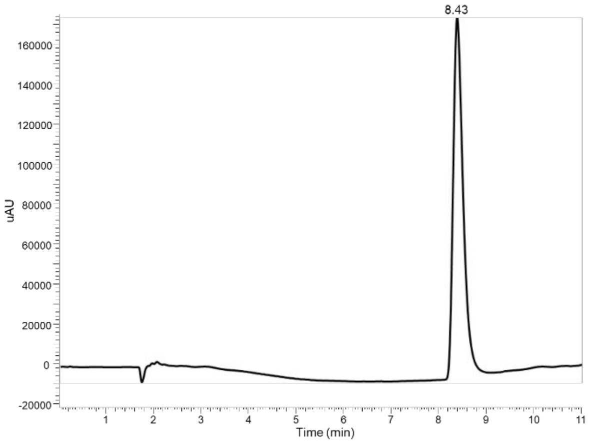

HPLC-UV profile of compound **10.**

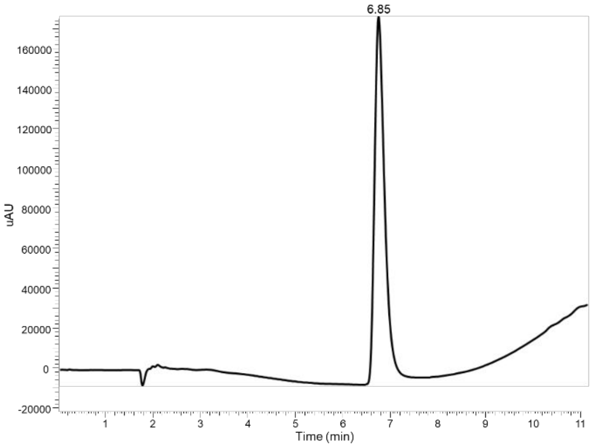

HPLC-UV profile of compound **13.**
